# Single-Particle Characterization of SARS-CoV-2 Isoelectric Point and Comparison to Variants of Interest

**DOI:** 10.3390/microorganisms9081606

**Published:** 2021-07-28

**Authors:** Oluwatoyin Areo, Pratik U. Joshi, Mark Obrenovich, Moncef Tayahi, Caryn L. Heldt

**Affiliations:** 1 Department of Chemical Engineering, Michigan Technological University, Houghton, MI 49931, USA; oareo@mtu.edu (O.A.); pjoshi1@mtu.edu (P.U.J.); 2 Health Research Institute, Michigan Technological University, Houghton, MI 49931, USA; 3 Louis Stokes Department of Veterans Affairs Medical Center, Department of Infectious Disease, Cleveland, OH 44106, USA; meo5@case.edu; 4 The Gilgamesh Foundation for Medical Science, Research and Education, Cleveland, OH 44116, USA; 5 Case Western Reserve University, Department of Chemistry, Cleveland, OH 44106, USA; 6 Department of Electrical Engineering and Computer Science, University of Cincinnati, Cincinnati, OH 45221, USA; moncef.tayahi@gmail.com

**Keywords:** emerging viruses, surface characterization, biophysics, adhesion, adsorption, dipole

## Abstract

SARS-CoV-2, the cause of COVID-19, is a new, highly pathogenic coronavirus, which is the third coronavirus to emerge in the past 2 decades and the first to become a global pandemic. The virus has demonstrated itself to be extremely transmissible and deadly. Recent data suggest that a targeted approach is key to mitigating infectivity. Due to the proliferation of cataloged protein and nucleic acid sequences in databases, the function of the nucleic acid, and genetic encoded proteins, we make predictions by simply aligning sequences and exploring their homology. Thus, similar amino acid sequences in a protein usually confer similar biochemical function, even from distal or unrelated organisms. To understand viral transmission and adhesion, it is key to elucidate the structural, surface, and functional properties of each viral protein. This is typically first modeled in highly pathogenic species by exploring folding, hydrophobicity, and isoelectric point (IEP). Recent evidence from viral RNA sequence modeling and protein crystals have been inadequate, which prevent full understanding of the IEP and other viral properties of SARS-CoV-2. We have thus experimentally determined the IEP of SARS-CoV-2. Our findings suggest that for enveloped viruses, such as SARS-CoV-2, estimates of IEP by the amino acid sequence alone may be unreliable. We compared the experimental IEP of SARS-CoV-2 to variants of interest (VOIs) using their amino acid sequence, thus providing a qualitative comparison of the IEP of VOIs.

The recent pandemic involving SARS-CoV-2, which causes COVID-19, represents serious and emerging threats worldwide. While the majority of COVID-19 cases are largely asymptomatic or mild clinical presentation, some can be severe or deadly in infected patients. Severe cases often develop acute respiratory distress syndrome and are deadly in spite of intubation, mechanical ventilation, and costly ICU care. Coronaviruses, such as SARS-CoV-2, are single-stranded, RNA macromolecules that have complex surface physicochemical properties, which give rise to their adsorption behavior. The adsorption of the virus to surfaces could give rise to increased transmission. BLAST and FASTA scans are typical search tools, which are performed on a nucleotide or amino acid sequence to impart structural information or predict the protein function. Information from other structural methods, such as protein crystallography, help to elucidate function and behavior. However, we sometimes find that the prediction of function and other features, such as isoelectric point (IEP), are not accurate and experimental measurements must be performed.

Understanding virus adsorption can help to facilitate safe practices. For example, learning how to repel viruses from surfaces or to adsorb them could be used to improve filtration devices and personal protective equipment. The physicochemical properties of the virus paired with environmental conditions facilitate virus adsorption [1,2]. The adhesion mechanism through which viruses are adsorbed is driven by electrostatic [3] and van der Waals interactions [4], as described by the extended Derjaguin–Landau–Verwey–Overbeek (XDLVO) model [5,6]. These interactions are controlled by environmental factors such as pH, temperature, and humidity [7]. Disrupting the adsorption of a virus to a surface can be achieved by manipulating the factors that contribute to the interactions.

Viruses have extremely complicated structures compared to proteins. One of the most prominent interactions for adsorption is electrostatic. While proteins can be described by their charge and IEP, which is the pH at which they are neutrally charged [8,9], this type of description is more difficult for viral particles. Nonenveloped viruses have a protein shell that folds into a large nanoparticle structure. However, enveloped viruses, such as SARS-CoV-2, have glycosylations and a lipid bilayer, making surface characterization much more difficult to predict and require experimental measurements.

Conventional methods for measuring virus IEP use bulk viral solutions. Zeta potential measures the electrostatic potential difference between the electric double layer surrounding the virus particle and the surrounding solution at the shear plane [10]. However, zeta potential requires a large volume of highly concentrated virus sample and is limited by virus solubility [11] and the presence of impurities [12]. Another IEP measurement is isoelectric focusing (IEF) [13] and capillary isoelectric focusing (CIEF) [14]. Both IEF methods require the fluorescent tagging of viruses, which requires pure, concentrated solutions [15]. Different methods are needed to measure virus IEP in natural solutions without high purity and concentration requirements.

We developed a single-particle method to measure the IEP of virus with an atomic force microscope (AFM). The technique, called chemical force microscopy (CFM), uses a functionalized AFM tip to measure the adhesion force of the functionalized AFM tip and the virus immobilized on a surface [16,17]. The adhesion is measured in different pH solutions, thus measuring a range of electrostatic interactions near the IEP. The IEP for the nonenveloped porcine parvovirus was found to be 4.8–5.1 [16] and this was found to be similar as the value determined by IEF of 5.0 [18]. However, when the IEP for the main surface protein was calculated with UniProtKB using the entire protein sequence, the IEP was determined to be 5.8 [16]. For the enveloped bovine viral diarrhea virus (BVDV), the discrepancy between the measured IEP value with CFM and the calculation of the IEP of the main spike protein was even greater, at 4.3–4.5 and 6.9, respectively [16]. Due to the post-translational modifications such as glycosylation, and the presence of a lipid membrane, it is more imperative that enveloped viruses have a measured IEP and do not use the calculated value from the spike protein amino acid sequence.

Since CFM is a new method to measure virus IEP, we are still learning about the nuances of this method. The AFM tips used must have a low spring constant (around 0.1 N/m), as the forces we are measuring are in the picoNewton range. We have also found that the magnitudes of the forces from the carboxylic acid probe are less than the quaternary amine [16,17]. This may be because the charge is permanent on the quaternary amine versus the carboxylic acid which is deprotonated at most pHs used in this work. We also noticed that the larger the force measured, the larger the spread in the histograms of the data for each data point. It is likely that the higher the force, the more sensitive the method is to the contact area of the probe to the virus. There are likely some force measurements that, instead of hitting the virus directly down, may only hit the side of the virus or the probe, thus reducing the force of that measurement since fewer molecules from the tip come into contact with the virus. As we study more viruses, we will continue to perfect this method.

The IEP of SARS-CoV-2 has been calculated in different ways and can be found in Table 1. The IEP ranges from 5.2–6.2. This is a large range when the goal is to either adsorb, trap, or repel the virus using electrostatic forces. The IEP values were obtained based on the identified proteins on SARS-CoV-2. The FASTA sequence was input into the Protparam tool from the Bioinformatics Resource Portal ExPASy [19] to obtain the values of the IEP based on the protein sequence. A major disadvantage is that calculated IEP values do not consider that some amino acids are buried when the protein folds and do not take into account any post-translational modifications. 

We used CFM to measure the IEP of SARS-CoV-2. Heat-inactivated and gamma-irradiated SARS-CoV-2 (USA-WA1/2020) isolates from BEI resources were covalently bound to a glass slide (see Figure 1a) and height analysis was performed on a Bruker Dimension ICON AFM with the ScanAsyst system (Santa Barbara, CA, USA) using a Bruker AC-40 AFM probe. The heat-inactivated virus contained many small particles (Figure S1) and was not further tested. The gamma-irradiated virus was immobilized on a glass slide, as described in Figure 1a. NT-MDT CSG10 gold-coated AFM probes were functionalized with thiol-C_12_ molecules that terminated in either a carboxylic acid (for a negatively-charged probe) or a quaternary amine (for a positively-charged probe). The adhesion force between the charged probe and the covalently bound virus using NHS/EDC chemistry was measured in 20 mM citrate buffer between pH 4–6 or 20 mM phosphate buffer at pH 7.0 with an AFM. By measuring a range of pHs, the IEP could be determined, as described previously [16,17], and is shown in Figure 1. The measured IEP for SARS-CoV-2 (USA-WA1/2020) was 5.2–5.3. This is on the low end of the IEPs from the calculated sources shown in Table 1. In our previous study using the enveloped virus BVDV, the CFM measurement was very different from the calculated IEP using the amino acid sequence [16]. We obtained a similar result with the enveloped SARS-CoV-2 in the present study. This trend may be evidence that, unlike nonenveloped viruses, calculated IEP may be less reliable for an enveloped virus than values obtained using CFM or another experimental method.

Since only the wild type (USA-WA1/2020, referred to as WT) could be obtained for experimental study, we compared the charge differences between two other variants of interest (VOI) that are currently circulating of SARS-CoV-2, B.1.1.7 (Alpha), and B.1.351 (Beta). The charge of the WT virus and its variants can be found in Figure 2. The mutations are found in different parts of the spike protein, including the receptor binding domain and the furin cleavage site [22].

The exact surface mutations of the WT and VOIs are found in Table 2. The charge density on the surface of the spike protein decreases in the VOIs. This would likely increase the IEP of the VOIs compared to the WT. The charge on the RBD was slightly higher for the WT compared to the two variants when 6VYB was used as the WT model (Table 2). However, the WT RBD charge was lower compared to the VOIs when 6VSB was used as a reference. Additionally, the E484K mutation made the B.1.351 variant slightly more positive compared to the B.1.1.7 variant. A similar effect was observed for surface hydrophobicity (Table S1). The surface hydrophobicity was calculated by applying the Eisenberg hydrophobicity scale for the surface-exposed residues [23] (Figure S3). The WT was more hydrophobic compared to the VOIs (Table S1). Between the two VOIs, B.1.351 was more hydrophobic than B.1.1.7.

**Figure 2:**
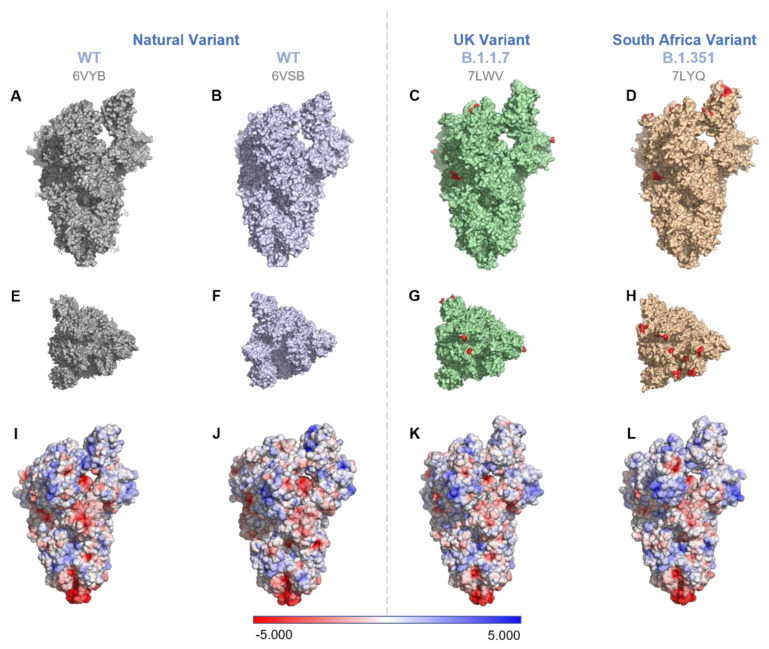
Comparison of the mutations (Rows 1 and 2) and surface electrostatic potentials (Row 3) on spike proteins of two VOIs with respect to the WT. WT (PDB ID: 6VYB [24] (1st column) and 6VSB [25] (2nd column)), B.1.1.7 (PDB ID: 7LWV [22]), and B.1.351 (PDB ID: 7LYQ [22]). (**A**–**D**) show the side view, 1st row; (**E–H**) show the top view, 2nd row. For the VOIs, the AA mutations on the S proteins (see Table 2 for details) are marked red with one residue before and after the actual mutational site for better visibility. (**I–L**) S-protein surface potentials of the WT and VOIs. The surface potentials were generated by preparing molecules with the pdb2pqr method and applying APBS electrostatics using PyMol v2.4.1.

The IEP of SARS-CoV-2 was determined using CFM. The IEP in 20 mM salt was 5.2–5.3. This is on the low end of values calculated from different amino acid sequences of the spike protein. Enveloped viruses contain glycosylation on their spike proteins that likely change the IEP, thus requiring a measured IEP compared to a calculated IEP. CFM is a novel method to measure virus IEP, which does not require high-purity and high-concentration virus stocks; it is a single-particle method that targets the virus particles individually for the measurement. Without access to VOIs of SARS-CoV-2, their sequence changes were used to calculate the change in surface charge and hydrophobicity compared to WT. The VOIs have a lower charge and lower hydrophobicity than the WT, and this may play a role in the increased transmission of the VOIs.

## Figures and Tables

**Figure 1:**
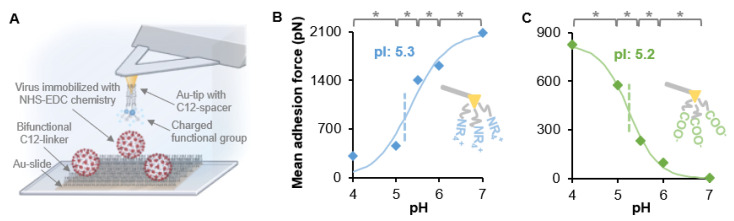
Isoelectric point determination using CFM. (**A**) Inactivated SARS-CoV-2 particles were covalently immobilized on a Au-coated slide that contained a self-assembled monolayer that presented COOH and CH_3_ functional groups. NHS/EDC chemistry covalently bound the virus to the COOH groups [16,17]. Au tips were functionalized to provide either negatively charged carboxyl (COO^−^) or positively charged quaternary amine (NR_4_^+^) groups. Changes in the mean adhesion forces were measured with respect to pH with (**B**) a NR_4_^+^ probe or (**C**) a COO^−^ probe. The data points of the mean adhesion force vs. pH were fit to a sigmoidal curve and the IEP was determined to be the infection point. Each data point represents 450 force curves and the histogram for each individual point can be found in Figure S2. * *p* < 0.05 from student’s *t*-test. Image A was made with BioRender.com.

**Table 1:** Summary of current IEP values for SARS-CoV-2 S protein.

Calculation/Method	Protein	IEP	Reference
Amino acid sequence	SARS-CoV-2 spike glycoprotein	5.9	[20]
ProtParam	SARS-CoV-2 spike glycoprotein	6.24	[21]
ProtParam	His-tagged SARS-CoV-2 RBD ^1^	8.91	[14]
CIEF	His-tagged SARS-CoV-2 RBD ^1^	7.36–9.85	[14]
CIEF	S1/S2 subunit with His-tag	4.41–5.87	[14]

^1^ RBD–receptor binding domain.

**Table 2:** Comparison of surface charge on S protein of the two VOI with the wild type at pH 7.0.

Pango Lineage	Name	Mutations	PDB	Surface Charge (Formal Charge)	Surface Charge (Partial Charge)	RBD Charge
WT (Natural variant)	USA/WA1/2020	-	6VYB	−20	−17	9
		-	6VSB	−26	−23	6
B.1.1.7 (UK variant)	20J/501Y.V1	Δ69/70 Δ144Y N501Y A570D D614G P681H	7LWV	−12	−9	7
B.1.351 (South Africa variant)	20H/501.V2	K417N E484K N501Y D614G	7LYQ	−7	−4	8

Surface charges are based on the solvent-accessible (SA) residues calculated in PyMol using APBS electrostatics. The boundary conditions used for single Debye–Huckel function were as follows: solute dielectric constant—2.000; solvent dielectric constant—78.000; ionic strength—150 mM; temperature—310 K. RBD charges were calculated by adding the partial charges of the SA resides in the 319–541 region of chain B for 6VYB, 7LWV, and 7LYQ or chain A for 6VSB. The chain consideration was based on the chain identifier that showed an upconfiguration of the RBD in PyMol.

## Data Availability

All data generated or analyzed during this study are included in this published article (and its supplementary information files).

## Supplementary Materials

The following are available online at https://www.mdpi.com/article/10.3390/microorganisms9081606/s1. Figure S1: Topographic images and height analysis. Figure S2: Adhesion and representative force–distance curves. Figure S3: Surface characteristics of S-proteins of WT and VOI. Table S1: Surface hydrophobicity for the WT and VOI; Raw Data.
