# Supplementary material for: Single-Particle Characterization of SARS-CoV-2 Isoelectric Point and Comparison to Variants of Interest
Source: Microorganisms. 2021 Jul 28;9(8):1606. doi: 10.3390/microorganisms9081606 (PMC8401476; doi:10.3390/microorganisms9081606)
Supplement: Supplementary file 1 [file microorganisms-09-01606-s001.zip › Areo SARS COV 2 IEP Raw data supplemental.pdf]

Single-Particle Characterization of SARS-CoV-2 Isoelectric Point and Comparison to Variants of Interest

(Raw Data Supplemental)

Oluwatoyin Areo[1,2], Pratik U. Joshi[1,2], Mark Obrenovich[3,4,5], Moncef Tayahi[6], and Caryn L. Heldt[1,2]*

[1] Department of Chemical Engineering, Michigan Technological University, Houghton, MI USA.
[2] Health Research Institute, Michigan Technological University, Houghton, MI
[3]Louis Stokes Department of Veterans Affairs Medical Center, Department of Infectious Disease, Cleveland, OH 44106 USA.
[4]The Gilgamesh Foundation for Medical Science, Research and Education, Cleveland, OH 44116 USA
[5]Case Western Reserve University, Department of Chemistry, Cleveland, OH USA.
[6]Unversity of Cincinnati, Department of Electrical Engineering and Computer Science, Cincinnati, OH 45221
*Corresponding author: 1400 Townsend Dr., Houghton, MI 49931, USA
heldt@mtu.edu, phone 906-487-1134

Table RD1. Mean peak adhesion forces between quaternary ammonium group (positive charge) and SARS-CoV-2 (USA-WA1/2020) at different pH used for Figure 1B. S1, S2, and S3 represent three separately immobilized solutions containing gamma irradiated virus stock.

S1	S2	S3
**pH 4**		
**Peak Force (pN)**		
654	436	276
637	321	271
637	344	270
591	367	283
683	235	288
0	229	257
384	161	246
545	172	249
459	115	253
470	138	266
258	115	277
407	132	272

S1	S2	S3
**pH 5**		
**Peak Force (pN)**		
395	285	263
384	452	250
384	514	259
420	489	412
361	452	366
393	463	403
400	501	374
409	523	392
341	519	402
375	480	383
417	516	278
371	378	344

S1	S2	S3
**pH 5.5**		
**Peak Force (pN)**		
867	1399	1936
934	1363	1936
969	1185	1767
906	1371	1718
913	1245	2190
928	1376	2386
852	1497	2186
939	1512	2362
951	1489	2364
900	1356	2237
839	1426	2179
900	1371	2170

S1	S2	S3
**pH 6**		
**Peak Force (pN)**		
1926	2955	1106
1191	2483	1114
1315	2553	1261
998	2522	1570
1261	2437	1632
2151	2684	1377
1772	2375	1478
1818	2421	1609
1949	2127	1176
2112	1841	1230
905	1965	1145
905	1493	1060

S1	S2	S3
**pH 7**		
**Peak Force (pN)**		
1900	117	1965
2513	2630	1878
2506	2338	1929
2579	2484	1834
2550	2367	1936
2433	2301	2433
2484	2163	2375
2382	2667	2280
2345	2674	2236
2185	2579	2177
2280	2579	2199
2046	2579	2323

333	293	291
390	270	279
402	321	292
516	315	322
717	298	362
562	161	310
533	138	333
602	132	285
23	178	251
0	0	253
252	407	258
69	344	244
0	270	251
562	281	221
522	465	224
545	264	222
390	178	223
505	241	166
556	178	145
539	143	120
568	75	151
551	195	151
447	155	118
465	0	32
442	247	206
482	86	106
396	120	95
442	109	66
476	149	201
459	80	194
396	304	253
493	384	194
568	356	174
344	447	208
430	321	201
402	178	192
304	115	212
361	132	145
120	161	190
0	195	188
350	161	154

362	486	356
366	440	492
393	412	544
325	437	501
294	389	554
306	423	529
291	408	525
324	395	535
375	420	532
371	310	501
356	322	559
374	210	455
359	38	455
392	266	458
340	285	458
437	170	492
384	306	396
301	278	343
219	279	474
201	545	417
132	544	381
198	562	471
171	483	457
598	535	434
528	529	445
573	526	467
532	501	463
567	485	496
547	539	431
544	520	460
553	557	392
553	539	366
523	528	356
439	526	244
449	563	324
501	508	301
448	525	228
375	517	226
482	551	219
492	597	210
520	576	195

923	1351	2192
860	1419	833
972	1373	941
865	1072	873
906	1376	820
893	1383	687
895	1306	880
801	1376	840
934	1341	812
928	1313	873
926	1431	888
949	1263	1413
926	1426	1401
873	1222	1508
911	1195	1493
873	1233	1355
888	1366	1504
1000	1358	1373
918	1202	1277
918	1539	1225
934	1283	1361
837	1534	1350
977	1311	1317
908	1489	1292
903	1688	1413
941	1336	1323
903	1313	1043
837	1182	1277
1142	1197	1223
1094	1685	1125
1129	1690	2600
1025	1741	2651
1010	1685	2696
1000	1494	880
964	1733	893
1061	1705	873
1099	1670	883
1231	1761	870
900	1791	827
1063	1426	903
974	1404	829

1215	1888	449
1114	1609	1385
2135	1725	1276
1996	1772	1075
2120	1191	990
1973	2561	1176
2491	2259	1129
2228	2104	843
2050	2367	789
2220	1949	928
2290	2646	959
2290	2808	1215
1485	2684	0
1632	2630	0
1888	2754	1385
1764	2816	959
1648	2847	395
1168	3288	433
1215	2955	456
1184	3141	449
1300	3063	565
1215	2785	348
503	2855	526
828	2483	805
603	1864	495
704	1679	534
1547	1919	921
1478	1640	255
1586	1423	774
1849	2050	774
1539	1748	1238
1346	1276	1052
1276	2004	2197
928	2855	2143
1601	3063	2251
1570	2994	2661
1663	2646	2437
1354	2638	2723
1524	2692	2568
1470	1733	2089
1369	1656	2120

2090	1622	2265
2133	1856	2192
1995	1395	2119
2046	1585	2513
1527	1330	2557
438	2112	2382
1301	2163	2513
2009	2250	2550
2090	102	2177
2689	285	2221
2608	2667	2126
2638	2681	2090
2652	2638	2163
2623	2630	183
2557	2550	2214
2681	848	22
2623	329	1973
2608	585	2075
2550	424	2185
2433	921	2031
2353	899	2068
2543	1081	2133
2470	1235	2170
2470	1279	869
2630	1242	913
2484	2499	964
2681	2440	979
2667	2470	1264
2711	2470	2338
2528	2499	2608
2470	2652	2506
2513	2535	2214
2484	2638	2265
2543	2659	2499
1176	2491	2389
1286	1834	2243
1257	2090	2272
1154	2002	2272
1366	1973	2470
2528	1768	2491
2543	2243	2382

40	115	14
6	0	0
0	109	23
17	132	120
17	206	45
0	178	11
11	579	2
80	356	11
212	413	181
0	516	192
229	493	210
166	218	174
201	120	81
0	206	59
241	310	38
212	178	54
258	333	0
115	252	43
120	252	0
0	465	18
69	212	68
247	465	199
201	505	244
184	367	201
229	218	267
178	0	260
247	327	167
218	264	154
281	275	147
161	327	154
229	333	181
287	482	217
258	597	242
275	447	273
247	396	228
218	574	154
166	189	251
281	275	108
235	270	226
270	252	334
275	264	260

526	539	269
498	499	256
399	511	297
389	562	279
372	514	347
359	513	434
359	511	454
542	517	451
576	251	433
482	198	494
471	231	371
551	239	442
387	245	389
409	235	446
350	231	408
356	247	470
374	279	477
542	269	513
607	567	485
575	573	528
658	542	556
669	528	547
615	591	489
560	544	510
619	554	499
603	575	495
612	578	470
114	508	468
458	700	483
460	731	467
437	696	468
405	663	517
403	658	449
567	671	443
564	717	508
591	709	426
604	569	452
643	547	392
594	499	458
652	502	440
629	464	467

926	1389	847
934	1338	949
913	1313	956
1018	1351	951
931	1391	979
944	1323	962
895	1275	946
796	1278	931
885	956	2326
875	1087	2360
822	1102	1090
822	1034	1094
878	931	1125
865	951	1078
883	976	1090
939	880	1054
1045	984	1061
832	1044	918
956	1472	902
956	1341	878
956	1383	1361
908	1253	1330
885	1509	1408
873	1391	1323
962	1416	1395
959	1373	1330
880	1396	1283
0	1361	1379
842	1300	1335
875	1371	1404
895	1389	2340
883	1351	2244
811	1434	2308
822	1321	2373
847	1419	2304
837	1376	2219
819	1361	2290
786	1283	2302
845	1487	2315
987	1409	2221
885	1293	2460

1323	1980	2120
1392	1694	2344
557	596	201
750	572	727
843	665	797
379	596	232
503	1122	224
1570	704	588
1586	1137	1013
1756	2104	1238
1694	2491	1501
1346	2460	0
1276	2375	1300
1168	2158	1044
1532	1439	681
1447	1385	1741
1671	1416	1122
1501	2452	526
1377	2398	1346
1586	2468	0
1810	2429	619
1037	2127	1114
828	990	1331
1145	719	1400
820	1462	696
1292	1849	1052
805	2375	549
874	2476	952
1547	2390	975
1238	2599	1037
1926	2476	449
1949	1741	642
2460	1725	689
2390	1787	704
3203	1934	812
2746	1238	1423
3040	1578	1184
1795	2058	1679
1857	1671	2104
1833	1663	2367
1849	1710	1246

2543	2192	658
2616	2250	2484
2703	1754	1659
2601	1717	1600
2652	168	1512
2645	2784	1593
2513	2718	1571
2280	2820	2258
2148	2842	2353
2455	2433	2323
2112	2477	2367
2243	2287	2375
2119	2214	2309
1812	2309	2389
1965	1739	2338
1900	1739	2309
1666	1878	2375
161	1914	2221
2703	1819	2901
2703	2155	2828
2725	2082	2733
2579	2433	2659
2718	2155	1819
2630	2228	1827
2616	2667	1702
2528	2689	1790
2630	2754	1629
2696	2477	1856
2550	2491	1987
2564	2017	1819
2484	1469	1936
2440	1797	1768
2280	1615	1673
2674	1819	1965
2579	2404	2163
2762	2703	2594
2776	2930	2543
2659	2477	2375
2550	2470	2389
2586	2586	2294
2594	2521	2287

218	447	269
195	338	296
264	0	298
80	384	237
298	0	95
293	241	280
281	252	217
258	264	194
275	229	172
252	258	179
315	356	185
275	321	79
304	304	0
350	252	233
0	0	190
287	488	34
333	539	57
361	511	258
0	522	233
356	511	190
333	293	34
229	327	57
264	350	258
333	298	442
298	178	436
356	631	402
402	608	407
270	614	367
384	619	379
126	201	390
402	224	327
333	229	344
270	195	419
304	224	361
373	447	407
287	476	338
235	430	298
505	442	338
252	447	379
465	367	310
465	247	333

638	533	423
690	494	460
452	492	457
383	535	436
427	412	0
476	451	479
439	467	470
471	585	483
445	609	451
544	597	449
590	625	448
563	612	454
579	559	514
638	570	445
476	600	476
554	541	0
483	567	426
544	332	378
566	403	402
461	399	403
499	281	403
517	278	405
535	375	412
480	387	434
427	356	442
436	304	380
411	325	520
483	451	573
440	427	584
430	476	572
548	446	544
572	465	538
584	499	505
582	489	513
556	489	468
393	420	474
522	474	380
417	675	332
494	705	383
389	650	396
421	675	359

1018	1426	2544
1010	1318	2502
916	1456	2707
977	1386	2654
944	1358	2531
972	1411	2268
964	1376	2308
2083	1700	2491
2003	1474	2411
2079	1587	3095
1956	1633	997
1925	1716	964
1970	1386	972
1981	1499	944
1760	1560	911
1595	1502	918
2622	1575	944
2531	1856	827
2569	1804	974
2567	1869	895
2542	1811	926
2522	1889	862
2422	1869	903
2288	1970	817
2326	1849	865
2812	1851	860
2745	1939	870
2738	1119	850
2636	1039	2373
2643	936	2389
2553	989	2438
2585	1092	2444
1776	1067	2302
2322	1107	2498
1876	1097	2259
1938	1072	2400
2048	1117	1491
2549	1771	1493
1918	1630	1520
2052	1710	1395
1831	1726	1417

921	1493	1640
1903	1594	1191
1416	1779	1331
1795	1485	1423
1408	1269	1903
1524	1416	1656
1501	1284	2035
1408	1323	1857
1671	2243	2127
1919	2228	3350
2151	2220	1741
2143	2205	2290
1934	2019	3419
2004	1160	1717
2050	1779	1679
2081	1926	1539
1988	1307	1748
1988	1060	1895
0	952	3125
2545	1021	1338
2174	1656	1586
2182	1501	3265
2243	1710	3002
1818	1408	3381
1679	1501	719
2019	1323	526
1547	1191	0
1462	1586	0
1354	1408	634
1238	1454	797
1617	1903	681
1864	1671	0
1447	1787	843
1532	1671	719
1454	1772	797
2630	1717	882
2197	1748	1091
1524	1640	936
1462	1710	928
2174	1632	0
2089	1671	3009

2484	2543	2185
2499	2601	2126
1147	2557	2112
1147	877	1900
1125	599	1805
1235	2550	2228
1286	935	2075
2725	848	2236
2681	2309	1987
2667	387	2199
2667	2214	2082
2594	2448	2258
2638	2506	2345
2703	2674	2185
2696	2652	2280
2681	2586	2170
2711	2506	2470
0	2564	2543
1527	2616	2250
1425	2769	2272
1374	2645	2484
2404	2623	2638
2455	2630	2535
2389	2762	2564
2221	2828	2360
2185	2601	2426
0	2696	2199
446	2725	2214
0	2543	2177
428	321	2126
481	336	2206
324	2740	2923
262	2667	3237
402	3017	3134
0	2002	3142
525	2908	2776
560	3902	2579
857	2521	2484
630	2638	2411
0	2411	2689
813	2484	2543

298	315	281
384	229	1496
344	321	1540
0	298	1455
402	333	1413
321	350	1491
327	235	1449
321	281	1502
384	602	1585
310	671	1770
0	619	1560
442	522	1670
413	637	1747
384	258	1364
419	252	1355

495	678	356
412	612	350
448	653	340
429	689	366
562	700	312
522	705	510
595	502	575
567	517	556
581	533	523
584	491	600
569	505	480
591	562	505
661	476	529
530	504	505
0	505	485

1687	1733	2039
2016	1562	2085
1331	1748	1983
1273	1680	2003
1356	1834	2097
1358	1741	1181
1205	1650	1092
1290	1643	1221
1321	1572	1214
1180	1519	945
1260	1600	2373
1160	1552	2377
1205	1519	1979
1207	1668	1217
1197	1492	1370

2096	1447	2893
1106	1625	2878
921	2298	3040
936	2321	2437
596	2630	1656
2476	2584	1872
2661	1895	1988
2267	944	2058
2220	735	2135
2321	1160	2120
1215	1903	1756
1284	1980	1949
2336	1973	1957
2375	1586	1934
2174	1996	2050

787	2579	2082
1014	2521	2068
962	2594	2046
542	2564	2097
393	2696	2206
70	2630	2148
61	2638	2228
726	1943	2112
1014	2258	2119
735	1783	2148
700	2024	2448
1233	2133	2601
1032	2594	2535
87	2579	2623
1049	2689	2521

Table RD2. Mean peak adhesion forces between carboxyl group (negative charge) and SARS-CoV-2 (USA-WA1/2020) at different pH used for Figure 1C. S1, S2, and S3 represent three separately immobilized solutions containing gamma irradiated virus stock.

pH 4 Peak Force (pN)			pH 5 Peak Force (pN)			pH 5.5 Peak Force (pN)			pH 6 Peak Force (pN)			pH 7 Peak Force (pN)		
S1	S2	S3	S1	S2	S3	S1	S2	S3	S1	S2	S3	S1	S2	S3
1395	754	848	390	499	317	326	262	28	77	0	96	7	0	0
1329	754	918	552	386	430	368	220	283	121	0	89	4	0	0
1403	824	933	231	239	244	467	106	177	108	2	108	5	0	0
1271	680	923	617	446	0	439	297	0	113	0	113	4	0	0
1139	680	863	223	191	300	305	64	0	94	0	123	2	0	0
867	709	739	491	126	674	78	220	220	0	0	66	9	0	0
1131	714	695	576	779	0	191	340	0	108	0	55	7	0	0
925	655	804	617	629	24	0	305	220	112	0	76	4	0	0
966	0	799	528	800	175	35	347	149	121	0	81	9	0	0
850	0	809	329	710	605	0	262	99	112	0	57	2	0	0
1940	709	645	621	296	605	262	234	0	102	72	13	9	0	0
1907	675	670	560	467	609	135	255	241	68	100	112	5	0	0
1692	700	600	0	150	637	106	319	0	121	81	125	14	0	0
2039	754	700	471	438	759	276	0	149	91	0	83	37	0	0
1890	957	635	787	382	852	227	205	0	28	66	55	9	0	0
537	819	754	487	686	605	220	0	28	117	134	87	27	0	0
611	868	754	467	869	483	276	368	241	136	140	93	11	0	0
545	843	824	820	812	511	0	220	57	163	155	110	7	0	0
504	883	824	414	698	475	269	326	7	200	98	23	7	0	0
545	888	759	658	629	479	163	0	312	157	94	96	4	0	0
1247	690	893	593	0	881	0	205	255	176	0	125	92	0	39
1222	645	685	856	365	471	127	319	99	115	0	125	7	0	37
1321	566	749	211	593	893	28	305	21	112	0	117	7	0	46
1337	888	853	142	467	670	382	170	0	102	15	134	5	0	35
1131	516	794	175	487	974	404	340	35	140	0	70	4	0	0
2378	833	933	300	564	589	510	382	142	123	93	113	16	0	0
2402	784	943	276	621	840	588	0	418	51	93	110	11	0	51
2295	749	948	414	653	897	687	113	241	64	0	121	7	0	46
2435	794	903	775	414	1035	489	559	382	55	76	106	11	0	0
2510	838	1007	808	735	739	496	397	248	62	62	130	9	0	0
974	1106	724	1051	589	211	418	198	319	81	85	102	9	0	0
1090	1065	744	820	467	239	418	177	0	96	106	102	7	0	0
958	958	843	382	414	438	446	368	0	104	93	108	5	0	0
1214	1131	838	637	528	686	0	0	170	0	87	142	5	0	0

1313	1032	799
1552	1131	809
1527	1098	665
1808	1106	675
1626	1156	729
1610	1197	759
504	0	744
594	1131	814
702	1337	829
537	1263	829
578	1445	799
1420	1445	784
1131	1387	819
1032	1065	814
949	1230	754
817	1321	938
0	1379	898
132	1040	923
33	1048	953
0	1073	1027
2378	1048	953
2320	1024	769
2576	1379	769
2576	1263	833
2633	1247	744
1461	1247	764
1469	1247	729
1197	1247	779
1370	1354	719
1230	1337	685
834	1263	590
883	1313	700
784	817	829
883	949	819
991	867	873
660	883	908
677	999	838
462	1139	933
611	1230	784
627	1164	789
1032	1172	923

706	605	881
0	625	0
844	520	540
154	507	694
110	613	235
0	625	227
134	856	0
597	796	487
434	881	690
524	877	718
357	974	694
166	824	158
118	929	223
175	869	1396
227	605	0
727	865	284
292	889	219
621	613	264
1047	613	256
982	909	284
1189	548	686
954	313	593
1372	0	451
589	227	0
284	511	244
560	268	284
629	268	150
467	390	260
446	459	0
357	658	252
455	430	0
244	341	540
706	479	373
532	934	231
641	897	479
645	698	317
296	856	235
645	686	264
426	446	0
495	714	300
300	946	589

319	120	0
375	411	0
389	0	368
0	0	297
404	0	78
368	191	0
0	227	0
496	333	177
191	262	241
0	609	0
375	567	269
113	482	135
715	411	361
305	411	0
0	312	85
290	297	135
220	198	177
255	0	21
220	71	0
0	234	0
78	0	191
354	163	191
312	305	227
305	262	0
220	460	191
368	418	269
0	354	0
425	220	113
333	170	64
439	156	212
241	354	0
333	0	142
248	375	135
248	510	326
85	0	354
397	312	397
241	319	389
0	276	0
305	453	474
149	453	446
156	397	517

83	77	95
85	100	119
121	110	113
0	125	115
83	130	127
66	70	142
83	21	89
102	32	121
127	2	0
96	0	0
147	0	0
104	134	36
19	147	0
0	132	60
62	138	66
47	138	0
70	176	132
72	155	66
76	180	76
64	161	66
49	153	45
68	77	79
81	72	98
125	85	70
93	125	0
112	85	0
59	149	0
98	112	0
64	89	0
98	104	9
87	53	94
104	43	125
112	23	102
108	40	77
72	30	40
30	32	77
210	93	100
0	100	87
215	106	115
193	112	113
189	140	100

5	0	21
7	0	0
9	0	0
5	0	0
7	0	0
5	0	0
5	0	0
19	0	0
62	0	0
30	0	0
57	0	0
69	0	0
7	0	0
5	0	0
2	0	0
7	0	0
78	0	0
5	0	0
4	0	0
7	0	0
87	0	0
83	0	0
48	0	0
69	0	0
7	0	0
9	0	0
4	0	19
19	121	0
4	0	0
5	0	0
23	0	0
16	0	0
9	18	0
7	14	0
5	0	28
9	0	0
7	0	0
5	0	0
4	0	0
2	0	0
12	0	0

776	1048	0
999	1156	0
636	1114	665
916	1015	734
0	1114	0
0	1271	511
8	826	0
0	793	0
0	817	585
462	867	0
520	759	0
388	0	283
528	0	729
520	58	695
1131	272	566
1057	41	625
1123	0	0
982	413	179
834	396	0
0	338	342
1544	545	417
1445	363	685
1395	776	799
1494	768	690
1436	809	635
2014	842	0
1659	669	660
1841	1304	749
1907	1271	719
1791	1205	704
0	1247	888
0	1346	556
347	322	516
305	256	595
297	322	571
1791	223	620
1824	347	630
1998	710	179
1907	726	660
2072	966	645
338	908	799

114	641	459
300	658	536
134	783	637
101	800	256
686	649	349
682	605	170
727	966	414
1003	41	698
905	0	434
0	0	402
406	260	211
662	0	272
674	199	0
284	101	625
187	414	1035
195	288	986
0	325	1019
166	653	1039
597	515	1270
613	564	1871
268	487	1611
459	597	1270
491	881	1477
0	852	1757
0	422	1051
0	767	844
483	763	909
438	856	796
296	621	820
487	674	471
613	816	467
1120	877	0
1209	710	414
1189	682	0
1226	568	345
572	787	556
942	771	0
722	0	77
1153	406	227
1246	0	187
483	426	235

205	503	524
651	276	659
0	333	135
595	354	411
460	0	241
184	28	701
389	375	234
14	0	78
283	71	0
0	135	290
120	340	467
120	184	0
57	227	382
255	347	503
0	262	397
361	163	234
305	227	184
312	241	460
489	0	368
156	297	411
425	411	276
389	0	467
319	177	347
354	227	276
326	227	567
538	156	418
0	227	177
538	290	170
276	0	382
333	0	432
241	248	297
0	241	297
127	319	191
0	220	241
297	0	184
0	21	177
0	276	312
14	0	397
113	305	227
220	21	340
0	283	375

223	64	168
144	76	70
93	115	115
64	83	17
140	98	110
62	112	40
212	138	0
202	138	0
164	89	176
200	108	123
225	70	113
123	62	112
193	93	191
142	93	0
142	106	0
140	117	0
123	112	0
91	108	0
106	132	0
129	121	0
112	117	140
47	121	134
45	151	113
113	127	77
87	138	47
104	181	189
193	155	98
193	153	159
191	189	113
153	140	163
189	68	0
91	55	0
89	0	0
151	96	0
45	62	0
70	170	110
291	138	19
208	166	43
225	119	76
195	130	102
189	164	142

9	0	0
7	0	0
7	0	0
4	0	0
4	0	0
7	25	0
7	0	0
7	0	0
5	0	0
9	0	0
5	0	0
7	0	0
7	0	0
5	0	0
32	18	0
9	0	0
9	0	0
9	18	0
9	0	0
4	0	0
5	0	0
4	0	0
7	0	0
2	0	0
4	0	0
9	0	103
7	0	0
5	0	0
9	0	0
9	0	0
7	0	0
5	0	0
37	0	0
9	0	0
4	0	0
7	0	51
5	0	50
11	0	44
9	0	43
9	0	46
9	0	41

90	776	546
233	1420	0
164	1106	427
63	1280	605
137	1304	387
365	1230	571
417	759	536
106	231	397
407	223	610
5	281	0
11	363	709
5	206	288
5	859	496
16	867	0
0	883	0
217	1271	447
0	1131	432
132	124	258
565	1255	0
481	793	0
407	1048	660
476	1032	0
523	1032	615
1258	982	650
1284	842	0
1437	958	967
1374	958	819
1363	1106	957
127	182	967
106	140	769
137	495	833
74	545	843
243	594	719
1178	537	784

459	377	256
313	1246	475
382	1477	0
625	1810	629
658	1818	467
994	1717	499
532	698	552
658	731	564
605	889	917
873	739	877
877	597	1076
901	929	921
751	873	917
763	978	1526
235	986	1384
0	877	856
203	613	1477
333	507	1376
1611	641	791
1538	605	808
2013	475	1197
1932	231	873
1688	524	869
0	101	341
357	406	0
414	231	451
308	1928	576
284	1680	714
0	1843	333
0	1761	223
0	1749	227
0	824	280
146	248	211
0	771	114

382	0	248
368	205	262
482	347	0
0	0	212
198	319	0
0	0	375
0	0	559
191	368	489
220	191	382
255	482	0
0	326	297
269	312	106
212	28	234
0	0	361
149	404	283
85	0	517
503	0	524
283	0	0
170	106	319
368	191	0
397	7	28
347	113	326
418	184	382
28	347	439
397	248	404
439	375	149
375	227	163
418	361	198
538	609	120
0	524	78
305	297	21
312	375	227
0	404	0
361	227	0

134	170	164
229	13	104
219	178	106
132	204	81
113	149	172
110	147	136
140	155	151
132	159	187
136	149	193
153	185	178
125	172	180
68	164	170
112	127	34
102	170	119
113	0	68
234	34	43
244	87	0
217	93	9
231	77	0
100	108	0
144	0	72
176	123	76
140	13	98
93	115	108
89	115	85
197	91	85
25	96	108
193	106	108
202	119	115
202	112	93
181	119	77
221	77	60
206	104	83
215	138	108

7	0	44
9	0	0
4	0	53
25	0	51
9	0	0
7	0	0
30	0	0
2	0	0
25	0	0
19	0	0
2	0	0
9	0	0
5	0	0
11	0	0
7	0	0
5	0	0
5	0	0
5	0	0
5	0	0
23	0	0
7	0	0
7	0	0
9	0	0
14	0	0
7	0	0
5	0	0
5	0	0
11	0	0
5	0	0
9	0	0
7	0	0
9	0	0
9	0	0
7	0	0

Table RD3: Surface Residues of 6VSB, 7LWV, and 7LYQ obtained from PyMol V2.4.1

6VSB								
chain	resi	resn	chain	resi	resn	chain	resi	resn
A	27	ALA	B	27	ALA	C	27	ALA
A	28	TYR	B	28	TYR	C	28	TYR
A	29	THR	B	29	THR	C	29	THR
A	30	ASN	B	30	ASN	C	30	ASN
A	32	PHE	B	31	SER	C	32	PHE
A	33	THR	B	32	PHE	C	33	THR
A	34	ARG	B	33	THR	C	34	ARG
A	38	TYR	B	34	ARG	C	36	VAL
A	39	PRO	B	37	TYR	C	37	TYR
A	40	ASP	B	38	TYR	C	38	TYR
A	41	LYS	B	39	PRO	C	39	PRO
A	42	VAL	B	40	ASP	C	40	ASP
A	44	ARG	B	41	LYS	C	41	LYS
A	45	SER	B	42	VAL	C	42	VAL
A	46	SER	B	44	ARG	C	43	PHE
A	47	VAL	B	45	SER	C	44	ARG
A	48	LEU	B	46	SER	C	45	SER
A	49	HIS	B	47	VAL	C	46	SER
A	50	SER	B	48	LEU	C	47	VAL
A	51	THR	B	49	HIS	C	48	LEU
A	52	GLN	B	50	SER	C	49	HIS
A	53	ASP	B	51	THR	C	50	SER
A	54	LEU	B	52	GLN	C	51	THR
A	57	PRO	B	53	ASP	C	52	GLN
A	58	PHE	B	54	LEU	C	53	ASP
A	59	PHE	B	56	LEU	C	54	LEU
A	60	SER	B	57	PRO	C	57	PRO
A	61	ASN	B	58	PHE	C	58	PHE
A	63	THR	B	59	PHE	C	59	PHE
A	64	TRP	B	60	SER	C	60	SER
A	65	PHE	B	61	ASN	C	61	ASN
A	66	HIS	B	63	THR	C	63	THR
A	79	PHE	B	64	TRP	C	64	TRP
A	80	ASP	B	65	PHE	C	65	PHE
A	81	ASN	B	66	HIS	C	66	HIS
A	82	PRO	B	81	ASN	C	80	ASP
A	83	VAL	B	82	PRO	C	81	ASN
A	84	LEU	B	83	VAL	C	82	PRO
A	85	PRO	B	84	LEU	C	83	VAL
A	87	ASN	B	85	PRO	C	84	LEU
A	88	ASP	B	87	ASN	C	85	PRO
A	92	PHE	B	88	ASP	C	86	PHE
A	94	SER	B	91	TYR	C	87	ASN
A	95	THR	B	92	PHE	C	88	ASP
A	99	ASN	B	94	SER	C	91	TYR
A	100	ILE	B	95	THR	C	93	ALA
A	101	ILE	B	96	GLU	C	94	SER
A	102	ARG	B	97	LYS	C	95	THR
A	104	TRP	B	98	SER	C	99	ASN
A	105	ILE	B	99	ASN	C	100	ILE
A	108	THR	B	100	ILE	C	101	ILE
A	109	THR	B	101	ILE	C	102	ARG
A	110	LEU	B	102	ARG	C	103	GLY
A	111	ASP	B	104	TRP	C	104	TRP
A	112	SER	B	105	ILE	C	105	ILE
A	113	LYS	B	108	THR	C	108	THR
A	114	THR	B	109	THR	C	109	THR
A	115	GLN	B	110	LEU	C	110	LEU
A	118	LEU	B	111	ASP	C	111	ASP
A	120	VAL	B	112	SER	C	112	SER
A	121	ASN	B	113	LYS	C	113	LYS
A	122	ASN	B	114	THR	C	114	THR
A	123	ALA	B	115	GLN	C	115	GLN
A	124	THR	B	118	LEU	C	116	SER
A	125	ASN	B	119	ILE	C	118	LEU
A	126	VAL	B	120	VAL	C	119	ILE
A	127	VAL	B	121	ASN	C	120	VAL
A	128	ILE	B	122	ASN	C	121	ASN
A	129	LYS	B	123	ALA	C	122	ASN
A	130	VAL	B	124	THR	C	123	ALA
A	132	GLU	B	125	ASN	C	124	THR
A	133	PHE	B	126	VAL	C	125	ASN
A	134	GLN	B	127	VAL	C	126	VAL
A	135	PHE	B	128	ILE	C	127	VAL
A	136	CYS	B	129	LYS	C	128	ILE
A	137	ASN	B	130	VAL	C	129	LYS
A	138	ASP	B	131	CYS	C	130	VAL
A	139	PRO	B	132	GLU	C	131	CYS
A	140	PHE	B	133	PHE	C	132	GLU
A	141	LEU	B	134	GLN	C	133	PHE
A	142	GLY	B	135	PHE	C	134	GLN
A	156	GLU	B	136	CYS	C	135	PHE
A	157	PHE	B	137	ASN	C	136	CYS
A	158	ARG	B	138	ASP	C	137	ASN
A	159	VAL	B	139	PRO	C	138	ASP

7LWV								
chain	resi	resn	chain	resi	resn	chain	resi	resn
A	27	ALA	B	27	ALA	C	27	ALA
A	28	TYR	B	28	TYR	C	28	TYR
A	29	THR	B	29	THR	C	30	ASN
A	30	ASN	B	30	ASN	C	32	PHE
A	32	PHE	B	32	PHE	C	33	THR
A	33	THR	B	33	THR	C	34	ARG
A	38	TYR	B	34	ARG	C	39	PRO
A	39	PRO	B	39	PRO	C	40	ASP
A	40	ASP	B	40	ASP	C	41	LYS
A	41	LYS	B	44	ARG	C	42	VAL
A	42	VAL	B	46	SER	C	44	ARG
A	44	ARG	B	47	VAL	C	45	SER
A	45	SER	B	48	LEU	C	46	SER
A	46	SER	B	49	HIS	C	47	VAL
A	47	VAL	B	50	SER	C	48	LEU
A	48	LEU	B	51	THR	C	49	HIS
A	49	HIS	B	52	GLN	C	50	SER
A	50	SER	B	53	ASP	C	51	THR
A	51	THR	B	54	LEU	C	52	GLN
A	52	GLN	B	56	LEU	C	53	ASP
A	53	ASP	B	57	PRO	C	54	LEU
A	54	LEU	B	58	PHE	C	57	PRO
A	57	PRO	B	59	PHE	C	58	PHE
A	58	PHE	B	60	SER	C	59	PHE
A	59	PHE	B	61	ASN	C	60	SER
A	60	SER	B	63	THR	C	61	ASN
A	61	ASN	B	64	TRP	C	63	THR
A	63	THR	B	65	PHE	C	64	TRP
A	64	TRP	B	66	HIS	C	65	PHE
A	65	PHE	B	81	ASN	C	66	HIS
A	66	HIS	B	82	PRO	C	80	ASP
A	79	PHE	B	83	VAL	C	81	ASN
A	80	ASP	B	84	LEU	C	82	PRO
A	82	PRO	B	85	PRO	C	83	VAL
A	83	VAL	B	87	ASN	C	84	LEU
A	84	LEU	B	88	ASP	C	85	PRO
A	85	PRO	B	95	THR	C	87	ASN
A	87	ASN	B	97	LYS	C	88	ASP
A	88	ASP	B	98	SER	C	94	SER
A	99	ASN	B	99	ASN	C	99	ASN
A	100	ILE	B	100	ILE	C	100	ILE
A	101	ILE	B	101	ILE	C	101	ILE
A	102	ARG	B	102	ARG	C	104	TRP
A	104	TRP	B	108	THR	C	105	ILE
A	108	THR	B	109	THR	C	108	THR
A	109	THR	B	111	ASP	C	109	THR
A	110	LEU	B	112	SER	C	110	LEU
A	111	ASP	B	113	LYS	C	111	ASP
A	112	SER	B	114	THR	C	112	SER
A	113	LYS	B	115	GLN	C	113	LYS
A	114	THR	B	118	LEU	C	114	THR
A	115	GLN	B	120	VAL	C	115	GLN
A	120	VAL	B	122	ASN	C	118	LEU
A	121	ASN	B	123	ALA	C	120	VAL
A	122	ASN	B	124	THR	C	121	ASN
A	123	ALA	B	125	ASN	C	122	ASN
A	124	THR	B	127	VAL	C	123	ALA
A	125	ASN	B	129	LYS	C	124	THR
A	126	VAL	B	132	GLU	C	125	ASN
A	127	VAL	B	133	PHE	C	127	VAL
A	129	LYS	B	134	GLN	C	130	VAL
A	130	VAL	B	135	PHE	C	132	GLU
A	132	GLU	B	136	CYS	C	133	PHE
A	134	GLN	B	137	ASN	C	134	GLN
A	135	PHE	B	138	ASP	C	135	PHE
A	136	CYS	B	140	PHE	C	136	CYS
A	137	ASN	B	141	LEU	C	137	ASN
A	138	ASP	B	155	SER	C	138	ASP
A	140	PHE	B	156	GLU	C	139	PRO
A	141	LEU	B	157	PHE	C	140	PHE
A	142	GLY	B	158	ARG	C	157	PHE
A	156	GLU	B	160	TYR	C	158	ARG
A	157	PHE	B	161	SER	C	159	VAL
A	158	ARG	B	162	SER	C	160	TYR
A	159	VAL	B	164	ASN	C	161	SER
A	160	TYR	B	165	ASN	C	162	SER
A	161	SER	B	166	CYS	C	163	ALA
A	162	SER	B	167	THR	C	164	ASN
A	163	ALA	B	168	PHE	C	165	ASN
A	164	ASN	B	169	GLU	C	166	CYS
A	165	ASN	B	170	TYR	C	167	THR
A	166	CYS	B	171	VAL	C	168	PHE
A	167	THR	B	172	SER	C	169	GLU
A	168	PHE	B	173	GLN	C	170	TYR
A	169	GLU	B	174	PRO	C	171	VAL

7LYQ								
chain	resi	resn	chain	resi	resn	chain	resi	resn
A	27	ALA	B	27	ALA	C	27	ALA
A	28	TYR	B	28	TYR	C	28	TYR
A	29	THR	B	29	THR	C	30	ASN
A	30	ASN	B	30	ASN	C	32	PHE
A	32	PHE	B	32	PHE	C	33	THR
A	33	THR	B	33	THR	C	34	ARG
A	38	TYR	B	34	ARG	C	39	PRO
A	39	PRO	B	39	PRO	C	40	ASP
A	40	ASP	B	40	ASP	C	41	LYS
A	41	LYS	B	46	SER	C	42	VAL
A	44	ARG	B	47	VAL	C	44	ARG
A	45	SER	B	48	LEU	C	45	SER
A	46	SER	B	49	HIS	C	46	SER
A	47	VAL	B	50	SER	C	47	VAL
A	48	LEU	B	51	THR	C	48	LEU
A	49	HIS	B	52	GLN	C	49	HIS
A	50	SER	B	53	ASP	C	50	SER
A	51	THR	B	54	LEU	C	51	THR
A	52	GLN	B	57	PRO	C	52	GLN
A	53	ASP	B	58	PHE	C	53	ASP
A	54	LEU	B	59	PHE	C	54	LEU
A	57	PRO	B	60	SER	C	57	PRO
A	58	PHE	B	61	ASN	C	58	PHE
A	59	PHE	B	63	THR	C	59	PHE
A	60	SER	B	64	TRP	C	60	SER
A	61	ASN	B	65	PHE	C	61	ASN
A	63	THR	B	66	HIS	C	63	THR
A	64	TRP	B	81	ASN	C	64	TRP
A	65	PHE	B	82	PRO	C	65	PHE
A	66	HIS	B	83	VAL	C	66	HIS
A	79	PHE	B	84	LEU	C	80	ASP
A	80	ASP	B	85	PRO	C	81	ASN
A	82	PRO	B	87	ASN	C	82	PRO
A	83	VAL	B	88	ASP	C	83	VAL
A	84	LEU	B	97	LYS	C	84	LEU
A	85	PRO	B	98	SER	C	85	PRO
A	87	ASN	B	99	ASN	C	87	ASN
A	88	ASP	B	100	ILE	C	88	ASP
A	99	ASN	B	108	THR	C	94	SER
A	100	ILE	B	109	THR	C	99	ASN
A	102	ARG	B	111	ASP	C	100	ILE
A	108	THR	B	112	SER	C	101	ILE
A	109	THR	B	113	LYS	C	104	TRP
A	111	ASP	B	114	THR	C	105	ILE
A	112	SER	B	115	GLN	C	108	THR
A	113	LYS	B	122	ASN	C	109	THR
A	114	THR	B	123	ALA	C	110	LEU
A	120	VAL	B	124	THR	C	111	ASP
A	122	ASN	B	125	ASN	C	112	SER
A	123	ALA	B	127	VAL	C	113	LYS
A	124	THR	B	129	LYS	C	114	THR
A	125	ASN	B	133	PHE	C	118	LEU
A	127	VAL	B	134	GLN	C	120	VAL
A	129	LYS	B	135	PHE	C	121	ASN
A	130	VAL	B	136	CYS	C	122	ASN
A	132	GLU	B	137	ASN	C	123	ALA
A	134	GLN	B	138	ASP	C	124	THR
A	135	PHE	B	140	PHE	C	125	ASN
A	136	CYS	B	141	LEU	C	127	VAL
A	137	ASN	B	155	SER	C	133	PHE
A	138	ASP	B	157	PHE	C	134	GLN
A	140	PHE	B	158	ARG	C	135	PHE
A	141	LEU	B	160	TYR	C	136	CYS
A	142	GLY	B	161	SER	C	137	ASN
A	156	GLU	B	162	SER	C	138	ASP
A	157	PHE	B	164	ASN	C	139	PRO
A	158	ARG	B	165	ASN	C	140	PHE
A	159	VAL	B	166	CYS	C	157	PHE
A	160	TYR	B	167	THR	C	158	ARG
A	161	SER	B	168	PHE	C	160	TYR
A	162	SER	B	169	GLU	C	161	SER
A	163	ALA	B	170	TYR	C	162	SER
A	164	ASN	B	171	VAL	C	163	ALA
A	165	ASN	B	172	SER	C	164	ASN
A	166	CYS	B	173	GLN	C	165	ASN
A	167	THR	B	174	PRO	C	166	CYS
A	168	PHE	B	175	PHE	C	167	THR
A	169	GLU	B	176	LEU	C	168	PHE
A	170	TYR	B	187	LYS	C	169	GLU
A	171	VAL	B	190	ARG	C	170	TYR
A	172	SER	B	196	ASN	C	171	VAL
A	173	GLN	B	197	ILE	C	172	SER
A	174	PRO	B	198	ASP	C	173	GLN
A	175	PHE	B	202	LYS	C	174	PRO
A	176	LEU	B	205	SER	C	175	PHE

A	160	TYR	B	140	PHE	C	139	PRO
A	161	SER	B	141	LEU	C	140	PHE
A	162	SER	B	155	SER	C	157	PHE
A	163	ALA	B	156	GLU	C	158	ARG
A	164	ASN	B	157	PHE	C	159	VAL
A	165	ASN	B	158	ARG	C	160	TYR
A	166	CYS	B	159	VAL	C	161	SER
A	167	THR	B	160	TYR	C	162	SER
A	168	PHE	B	161	SER	C	163	ALA
A	169	GLU	B	162	SER	C	164	ASN
A	170	TYR	B	163	ALA	C	165	ASN
A	171	VAL	B	164	ASN	C	166	CYS
A	172	SER	B	165	ASN	C	167	THR
A	173	GLN	B	166	CYS	C	168	PHE
A	174	PRO	B	167	THR	C	169	GLU
A	175	PHE	B	168	PHE	C	170	TYR
A	176	LEU	B	169	GLU	C	171	VAL
A	187	LYS	B	170	TYR	C	172	SER
A	188	ASN	B	171	VAL	C	173	GLN
A	189	LEU	B	172	SER	C	174	PRO
A	190	ARG	B	173	GLN	C	175	PHE
A	192	PHE	B	174	PRO	C	176	LEU
A	195	LYS	B	175	PHE	C	187	LYS
A	196	ASN	B	176	LEU	C	188	ASN
A	197	ILE	B	187	LYS	C	189	LEU
A	198	ASP	B	188	ASN	C	190	ARG
A	199	GLY	B	189	LEU	C	192	PHE
A	200	TYR	B	190	ARG	C	194	PHE
A	202	LYS	B	192	PHE	C	195	LYS
A	203	ILE	B	194	PHE	C	196	ASN
A	204	TYR	B	195	LYS	C	197	ILE
A	205	SER	B	196	ASN	C	198	ASP
A	206	LYS	B	197	ILE	C	199	GLY
A	207	HIS	B	198	ASP	C	200	TYR
A	208	THR	B	199	GLY	C	201	PHE
A	209	PRO	B	200	TYR	C	202	LYS
A	210	ILE	B	201	PHE	C	203	ILE
A	211	ASN	B	202	LYS	C	204	TYR
A	212	LEU	B	203	ILE	C	205	SER
A	213	VAL	B	204	TYR	C	206	LYS
A	214	ARG	B	205	SER	C	207	HIS
A	215	ASP	B	206	LYS	C	208	THR
A	216	LEU	B	207	HIS	C	209	PRO
A	217	PRO	B	208	THR	C	210	ILE
A	218	GLN	B	209	PRO	C	211	ASN
A	219	GLY	B	217	PRO	C	212	LEU
A	220	PHE	B	218	GLN	C	213	VAL
A	221	SER	B	219	GLY	C	214	ARG
A	222	ALA	B	220	PHE	C	215	ASP
A	224	GLU	B	221	SER	C	216	LEU
A	225	PRO	B	222	ALA	C	217	PRO
A	226	LEU	B	224	GLU	C	218	GLN
A	227	VAL	B	225	PRO	C	219	GLY
A	228	ASP	B	226	LEU	C	220	PHE
A	230	PRO	B	227	VAL	C	221	SER
A	231	ILE	B	228	ASP	C	222	ALA
A	232	GLY	B	229	LEU	C	223	LEU
A	233	ILE	B	230	PRO	C	224	GLU
A	234	ASN	B	231	ILE	C	225	PRO
A	235	ILE	B	232	GLY	C	226	LEU
A	236	THR	B	233	ILE	C	227	VAL
A	237	ARG	B	234	ASN	C	228	ASP
A	239	GLN	B	235	ILE	C	230	PRO
A	242	LEU	B	236	THR	C	231	ILE
A	243	ALA	B	237	ARG	C	232	GLY
A	244	LEU	B	239	GLN	C	233	ILE
A	245	HIS	B	240	THR	C	234	ASN
A	246	ARG	B	241	LEU	C	235	ILE
A	261	GLY	B	242	LEU	C	236	THR
A	262	ALA	B	263	ALA	C	237	ARG
A	263	ALA	B	264	ALA	C	239	GLN
A	264	ALA	B	266	TYR	C	240	THR
A	265	TYR	B	267	VAL	C	241	LEU
A	266	TYR	B	268	GLY	C	242	LEU
A	267	VAL	B	269	TYR	C	243	ALA
A	269	TYR	B	270	LEU	C	244	LEU
A	270	LEU	B	271	GLN	C	245	HIS
A	271	GLN	B	272	PRO	C	261	GLY
A	272	PRO	B	273	ARG	C	262	ALA
A	273	ARG	B	274	THR	C	263	ALA
A	274	THR	B	278	LYS	C	264	ALA
A	278	LYS	B	279	TYR	C	265	TYR
A	279	TYR	B	280	ASN	C	266	TYR
A	280	ASN	B	281	GLU	C	267	VAL
A	281	GLU	B	282	ASN	C	268	GLY
A	282	ASN	B	283	GLY	C	269	TYR
A	283	GLY	B	284	THR	C	270	LEU
A	284	THR	B	285	ILE	C	271	GLN
A	285	ILE	B	286	THR	C	272	PRO
A	286	THR	B	287	ASP	C	273	ARG

A	170	TYR	B	175	PHE	C	172	SER
A	171	VAL	B	176	LEU	C	173	GLN
A	172	SER	B	187	LYS	C	174	PRO
A	173	GLN	B	190	ARG	C	175	PHE
A	174	PRO	B	196	ASN	C	176	LEU
A	175	PHE	B	197	ILE	C	187	LYS
A	176	LEU	B	198	ASP	C	188	ASN
A	187	LYS	B	199	GLY	C	192	PHE
A	188	ASN	B	202	LYS	C	196	ASN
A	190	ARG	B	204	TYR	C	198	ASP
A	192	PHE	B	205	SER	C	200	TYR
A	195	LYS	B	206	LYS	C	202	LYS
A	196	ASN	B	207	HIS	C	204	TYR
A	197	ILE	B	208	THR	C	206	LYS
A	198	ASP	B	209	PRO	C	207	HIS
A	199	GLY	B	217	PRO	C	208	THR
A	200	TYR	B	218	GLN	C	209	PRO
A	202	LYS	B	219	GLY	C	210	ILE
A	206	LYS	B	220	PHE	C	211	ASN
A	207	HIS	B	221	SER	C	212	LEU
A	208	THR	B	222	ALA	C	213	VAL
A	209	PRO	B	224	GLU	C	214	ARG
A	210	ILE	B	225	PRO	C	215	ASP
A	211	ASN	B	226	LEU	C	217	PRO
A	212	LEU	B	227	VAL	C	218	GLN
A	213	VAL	B	228	ASP	C	219	GLY
A	214	ARG	B	230	PRO	C	220	PHE
A	217	PRO	B	231	ILE	C	221	SER
A	218	GLN	B	232	GLY	C	224	GLU
A	219	GLY	B	233	ILE	C	225	PRO
A	220	PHE	B	234	ASN	C	226	LEU
A	221	SER	B	235	ILE	C	227	VAL
A	224	GLU	B	236	THR	C	228	ASP
A	225	PRO	B	237	ARG	C	229	LEU
A	226	LEU	B	239	GLN	C	230	PRO
A	227	VAL	B	242	LEU	C	231	ILE
A	228	ASP	B	263	ALA	C	232	GLY
A	230	PRO	B	264	ALA	C	233	ILE
A	231	ILE	B	266	TYR	C	234	ASN
A	234	ASN	B	267	VAL	C	235	ILE
A	235	ILE	B	268	GLY	C	236	THR
A	236	THR	B	269	TYR	C	241	LEU
A	237	ARG	B	270	LEU	C	242	LEU
A	239	GLN	B	271	GLN	C	243	ALA
A	242	LEU	B	272	PRO	C	244	LEU
A	243	ALA	B	273	ARG	C	245	HIS
A	244	LEU	B	274	THR	C	261	GLY
A	245	HIS	B	278	LYS	C	262	ALA
A	246	ARG	B	280	ASN	C	263	ALA
A	261	GLY	B	281	GLU	C	265	TYR
A	262	ALA	B	282	ASN	C	269	TYR
A	263	ALA	B	284	THR	C	270	LEU
A	269	TYR	B	286	THR	C	271	GLN
A	270	LEU	B	287	ASP	C	272	PRO
A	271	GLN	B	288	ALA	C	273	ARG
A	272	PRO	B	291	CYS	C	274	THR
A	273	ARG	B	292	ALA	C	278	LYS
A	274	THR	B	293	LEU	C	280	ASN
A	278	LYS	B	294	ASP	C	281	GLU
A	280	ASN	B	295	PRO	C	282	ASN
A	281	GLU	B	296	LEU	C	284	THR
A	282	ASN	B	297	SER	C	285	ILE
A	284	THR	B	298	GLU	C	286	THR
A	286	THR	B	300	LYS	C	287	ASP
A	287	ASP	B	301	CYS	C	288	ALA
A	288	ALA	B	302	THR	C	289	VAL
A	291	CYS	B	303	LEU	C	291	CYS
A	292	ALA	B	304	LYS	C	292	ALA
A	293	LEU	B	305	SER	C	293	LEU
A	294	ASP	B	306	PHE	C	294	ASP
A	295	PRO	B	307	THR	C	295	PRO
A	296	LEU	B	309	GLU	C	296	LEU
A	297	SER	B	310	LYS	C	297	SER
A	298	GLU	B	311	GLY	C	298	GLU
A	300	LYS	B	312	ILE	C	300	LYS
A	302	THR	B	313	TYR	C	301	CYS
A	303	LEU	B	314	GLN	C	302	THR
A	304	LYS	B	315	THR	C	303	LEU
A	305	SER	B	316	SER	C	304	LYS
A	306	PHE	B	317	ASN	C	305	SER
A	307	THR	B	318	PHE	C	306	PHE
A	309	GLU	B	319	ARG	C	307	THR
A	310	LYS	B	320	VAL	C	309	GLU
A	311	GLY	B	321	GLN	C	310	LYS
A	312	ILE	B	322	PRO	C	311	GLY
A	313	TYR	B	323	THR	C	312	ILE
A	314	GLN	B	324	GLU	C	313	TYR
A	315	THR	B	325	SER	C	314	GLN
A	316	SER	B	327	VAL	C	315	THR
A	317	ASN	B	328	ARG	C	316	SER

A	187	LYS	B	206	LYS	C	176	LEU
A	188	ASN	B	207	HIS	C	187	LYS
A	195	LYS	B	208	THR	C	188	ASN
A	196	ASN	B	209	PRO	C	192	PHE
A	197	ILE	B	217	PRO	C	196	ASN
A	198	ASP	B	218	GLN	C	198	ASP
A	199	GLY	B	219	GLY	C	200	TYR
A	200	TYR	B	220	PHE	C	202	LYS
A	202	LYS	B	221	SER	C	204	TYR
A	206	LYS	B	222	ALA	C	206	LYS
A	207	HIS	B	224	GLU	C	207	HIS
A	208	THR	B	225	PRO	C	208	THR
A	209	PRO	B	226	LEU	C	209	PRO
A	210	ILE	B	227	VAL	C	211	ASN
A	211	ASN	B	228	ASP	C	212	LEU
A	212	LEU	B	230	PRO	C	214	ARG
A	213	VAL	B	231	ILE	C	215	ASP
A	214	ARG	B	233	ILE	C	217	PRO
A	217	PRO	B	234	ASN	C	218	GLN
A	218	GLN	B	235	ILE	C	219	GLY
A	219	GLY	B	236	THR	C	220	PHE
A	220	PHE	B	237	ARG	C	221	SER
A	221	SER	B	239	GLN	C	224	GLU
A	224	GLU	B	242	LEU	C	225	PRO
A	225	PRO	B	263	ALA	C	226	LEU
A	226	LEU	B	264	ALA	C	227	VAL
A	227	VAL	B	269	TYR	C	228	ASP
A	228	ASP	B	270	LEU	C	229	LEU
A	231	ILE	B	271	GLN	C	230	PRO
A	233	ILE	B	272	PRO	C	231	ILE
A	234	ASN	B	273	ARG	C	232	GLY
A	235	ILE	B	274	THR	C	233	ILE
A	236	THR	B	278	LYS	C	234	ASN
A	237	ARG	B	280	ASN	C	235	ILE
A	239	GLN	B	281	GLU	C	236	THR
A	243	ALA	B	282	ASN	C	241	LEU
A	244	LEU	B	284	THR	C	242	LEU
A	245	HIS	B	286	THR	C	244	LEU
A	246	ARG	B	287	ASP	C	245	HIS
A	261	GLY	B	288	ALA	C	261	GLY
A	262	ALA	B	291	CYS	C	262	ALA
A	263	ALA	B	292	ALA	C	263	ALA
A	269	TYR	B	293	LEU	C	265	TYR
A	271	GLN	B	294	ASP	C	269	TYR
A	272	PRO	B	295	PRO	C	270	LEU
A	273	ARG	B	296	LEU	C	271	GLN
A	274	THR	B	298	GLU	C	272	PRO
A	278	LYS	B	300	LYS	C	273	ARG
A	280	ASN	B	302	THR	C	274	THR
A	281	GLU	B	303	LEU	C	278	LYS
A	282	ASN	B	304	LYS	C	280	ASN
A	284	THR	B	305	SER	C	281	GLU
A	286	THR	B	306	PHE	C	282	ASN
A	287	ASP	B	307	THR	C	284	THR
A	288	ALA	B	309	GLU	C	285	ILE
A	291	CYS	B	310	LYS	C	286	THR
A	292	ALA	B	311	GLY	C	287	ASP
A	293	LEU	B	312	ILE	C	288	ALA
A	294	ASP	B	313	TYR	C	289	VAL
A	295	PRO	B	314	GLN	C	291	CYS
A	297	SER	B	315	THR	C	292	ALA
A	298	GLU	B	316	SER	C	293	LEU
A	300	LYS	B	317	ASN	C	294	ASP
A	302	THR	B	318	PHE	C	295	PRO
A	303	LEU	B	319	ARG	C	296	LEU
A	304	LYS	B	320	VAL	C	297	SER
A	305	SER	B	321	GLN	C	298	GLU
A	306	PHE	B	322	PRO	C	300	LYS
A	307	THR	B	323	THR	C	301	CYS
A	309	GLU	B	324	GLU	C	302	THR
A	310	LYS	B	325	SER	C	303	LEU
A	311	GLY	B	327	VAL	C	304	LYS
A	312	ILE	B	328	ARG	C	305	SER
A	313	TYR	B	330	PRO	C	306	PHE
A	314	GLN	B	331	ASN	C	307	THR
A	315	THR	B	332	ILE	C	308	VAL
A	316	SER	B	333	THR	C	309	GLU
A	317	ASN	B	334	ASN	C	310	LYS
A	318	PHE	B	335	LEU	C	311	GLY
A	319	ARG	B	336	CYS	C	312	ILE
A	320	VAL	B	337	PRO	C	313	TYR
A	321	GLN	B	339	GLY	C	314	GLN
A	322	PRO	B	340	GLU	C	315	THR
A	323	THR	B	341	VAL	C	316	SER
A	324	GLU	B	342	PHE	C	317	ASN
A	325	SER	B	343	ASN	C	318	PHE
A	327	VAL	B	344	ALA	C	319	ARG
A	328	ARG	B	345	THR	C	320	VAL
A	335	LEU	B	346	ARG	C	321	GLN
A	336	CYS	B	347	PHE	C	322	PRO

A	287	ASP	B	288	ALA	C	274	THR
A	288	ALA	B	289	VAL	C	275	PHE
A	289	VAL	B	290	ASP	C	278	LYS
A	291	CYS	B	291	CYS	C	279	TYR
A	292	ALA	B	292	ALA	C	280	ASN
A	293	LEU	B	293	LEU	C	281	GLU
A	294	ASP	B	294	ASP	C	282	ASN
A	295	PRO	B	295	PRO	C	284	THR
A	296	LEU	B	296	LEU	C	285	ILE
A	297	SER	B	297	SER	C	286	THR
A	298	GLU	B	298	GLU	C	287	ASP
A	300	LYS	B	300	LYS	C	288	ALA
A	301	CYS	B	301	CYS	C	289	VAL
A	302	THR	B	302	THR	C	290	ASP
A	303	LEU	B	303	LEU	C	291	CYS
A	304	LYS	B	304	LYS	C	292	ALA
A	305	SER	B	305	SER	C	293	LEU
A	306	PHE	B	306	PHE	C	294	ASP
A	307	THR	B	307	THR	C	295	PRO
A	308	VAL	B	309	GLU	C	296	LEU
A	309	GLU	B	310	LYS	C	297	SER
A	310	LYS	B	311	GLY	C	298	GLU
A	311	GLY	B	312	ILE	C	300	LYS
A	312	ILE	B	313	TYR	C	301	CYS
A	313	TYR	B	314	GLN	C	302	THR
A	314	GLN	B	315	THR	C	303	LEU
A	315	THR	B	316	SER	C	304	LYS
A	316	SER	B	317	ASN	C	305	SER
A	317	ASN	B	318	PHE	C	306	PHE
A	318	PHE	B	319	ARG	C	307	THR
A	319	ARG	B	320	VAL	C	309	GLU
A	320	VAL	B	321	GLN	C	310	LYS
A	321	GLN	B	322	PRO	C	311	GLY
A	322	PRO	B	323	THR	C	312	ILE
A	323	THR	B	324	GLU	C	313	TYR
A	324	GLU	B	325	SER	C	314	GLN
A	325	SER	B	327	VAL	C	315	THR
A	326	ILE	B	328	ARG	C	316	SER
A	327	VAL	B	329	PHE	C	317	ASN
A	328	ARG	B	330	PRO	C	318	PHE
A	335	LEU	B	331	ASN	C	319	ARG
A	336	CYS	B	332	ILE	C	320	VAL
A	337	PRO	B	333	THR	C	321	GLN
A	338	PHE	B	334	ASN	C	322	PRO
A	339	GLY	B	335	LEU	C	323	THR
A	340	GLU	B	336	CYS	C	324	GLU
A	341	VAL	B	337	PRO	C	325	SER
A	342	PHE	B	338	PHE	C	327	VAL
A	343	ASN	B	339	GLY	C	328	ARG
A	344	ALA	B	340	GLU	C	329	PHE
A	345	THR	B	342	PHE	C	330	PRO
A	346	ARG	B	343	ASN	C	331	ASN
A	347	PHE	B	344	ALA	C	332	ILE
A	348	ALA	B	345	THR	C	333	THR
A	349	SER	B	346	ARG	C	334	ASN
A	351	TYR	B	347	PHE	C	335	LEU
A	352	ALA	B	348	ALA	C	336	CYS
A	353	TRP	B	349	SER	C	337	PRO
A	354	ASN	B	351	TYR	C	338	PHE
A	355	ARG	B	352	ALA	C	339	GLY
A	356	LYS	B	353	TRP	C	340	GLU
A	357	ARG	B	354	ASN	C	341	VAL
A	358	ILE	B	355	ARG	C	342	PHE
A	359	SER	B	356	LYS	C	343	ASN
A	360	ASN	B	357	ARG	C	344	ALA
A	361	CYS	B	358	ILE	C	345	THR
A	362	VAL	B	359	SER	C	346	ARG
A	364	ASP	B	360	ASN	C	347	PHE
A	365	TYR	B	362	VAL	C	348	ALA
A	366	SER	B	364	ASP	C	349	SER
A	367	VAL	B	366	SER	C	350	VAL
A	368	LEU	B	367	VAL	C	351	TYR
A	369	TYR	B	368	LEU	C	352	ALA
A	370	ASN	B	369	TYR	C	353	TRP
A	371	SER	B	370	ASN	C	354	ASN
A	372	ALA	B	371	SER	C	355	ARG
A	373	SER	B	372	ALA	C	356	LYS
A	374	PHE	B	373	SER	C	357	ARG
A	375	SER	B	374	PHE	C	358	ILE
A	376	THR	B	375	SER	C	359	SER
A	377	PHE	B	376	THR	C	360	ASN
A	378	LYS	B	377	PHE	C	361	CYS
A	379	CYS	B	378	LYS	C	362	VAL
A	380	TYR	B	379	CYS	C	364	ASP
A	381	GLY	B	380	TYR	C	366	SER
A	382	VAL	B	381	GLY	C	367	VAL
A	383	SER	B	382	VAL	C	368	LEU
A	384	PRO	B	384	PRO	C	369	TYR
A	385	THR	B	385	THR	C	370	ASN
A	386	LYS	B	386	LYS	C	371	SER

A	318	PHE	B	330	PRO	C	317	ASN
A	319	ARG	B	331	ASN	C	318	PHE
A	320	VAL	B	332	ILE	C	319	ARG
A	321	GLN	B	333	THR	C	320	VAL
A	322	PRO	B	334	ASN	C	321	GLN
A	323	THR	B	335	LEU	C	322	PRO
A	324	GLU	B	336	CYS	C	323	THR
A	325	SER	B	337	PRO	C	324	GLU
A	327	VAL	B	339	GLY	C	325	SER
A	328	ARG	B	340	GLU	C	327	VAL
A	335	LEU	B	341	VAL	C	330	PRO
A	336	CYS	B	342	PHE	C	331	ASN
A	337	PRO	B	343	ASN	C	332	ILE
A	338	PHE	B	344	ALA	C	333	THR
A	339	GLY	B	345	THR	C	334	ASN
A	340	GLU	B	346	ARG	C	335	LEU
A	341	VAL	B	347	PHE	C	336	CYS
A	342	PHE	B	348	ALA	C	337	PRO
A	343	ASN	B	349	SER	C	339	GLY
A	344	ALA	B	351	TYR	C	340	GLU
A	345	THR	B	352	ALA	C	341	VAL
A	346	ARG	B	353	TRP	C	342	PHE
A	347	PHE	B	354	ASN	C	343	ASN
A	348	ALA	B	355	ARG	C	344	ALA
A	349	SER	B	356	LYS	C	345	THR
A	351	TYR	B	357	ARG	C	346	ARG
A	352	ALA	B	358	ILE	C	347	PHE
A	353	TRP	B	359	SER	C	349	SER
A	354	ASN	B	360	ASN	C	352	ALA
A	355	ARG	B	361	CYS	C	354	ASN
A	356	LYS	B	364	ASP	C	355	ARG
A	357	ARG	B	366	SER	C	356	LYS
A	358	ILE	B	367	VAL	C	357	ARG
A	359	SER	B	369	TYR	C	359	SER
A	360	ASN	B	370	ASN	C	360	ASN
A	362	VAL	B	371	SER	C	362	VAL
A	366	SER	B	372	ALA	C	364	ASP
A	367	VAL	B	373	SER	C	366	SER
A	368	LEU	B	374	PHE	C	367	VAL
A	369	TYR	B	375	SER	C	369	TYR
A	370	ASN	B	376	THR	C	370	ASN
A	371	SER	B	377	PHE	C	371	SER
A	372	ALA	B	378	LYS	C	372	ALA
A	373	SER	B	379	CYS	C	373	SER
A	374	PHE	B	380	TYR	C	374	PHE
A	375	SER	B	382	VAL	C	375	SER
A	376	THR	B	384	PRO	C	376	THR
A	377	PHE	B	385	THR	C	377	PHE
A	378	LYS	B	386	LYS	C	378	LYS
A	379	CYS	B	388	ASN	C	379	CYS
A	380	TYR	B	389	ASP	C	380	TYR
A	383	SER	B	390	LEU	C	381	GLY
A	384	PRO	B	391	CYS	C	382	VAL
A	385	THR	B	392	PHE	C	384	PRO
A	386	LYS	B	393	THR	C	385	THR
A	388	ASN	B	394	ASN	C	386	LYS
A	389	ASP	B	399	SER	C	388	ASN
A	390	LEU	B	402	ILE	C	389	ASP
A	392	PHE	B	403	ARG	C	390	LEU
A	393	THR	B	404	GLY	C	393	THR
A	394	ASN	B	405	ASP	C	399	SER
A	396	TYR	B	406	GLU	C	404	GLY
A	397	ALA	B	407	VAL	C	405	ASP
A	399	SER	B	408	ARG	C	407	VAL
A	403	ARG	B	409	GLN	C	408	ARG
A	404	GLY	B	410	ILE	C	409	GLN
A	405	ASP	B	411	ALA	C	411	ALA
A	406	GLU	B	412	PRO	C	412	PRO
A	407	VAL	B	413	GLY	C	413	GLY
A	408	ARG	B	414	GLN	C	414	GLN
A	409	GLN	B	415	THR	C	415	THR
A	410	ILE	B	416	GLY	C	416	GLY
A	411	ALA	B	417	LYS	C	417	LYS
A	412	PRO	B	418	ILE	C	420	ASP
A	413	GLY	B	419	ALA	C	421	TYR
A	414	GLN	B	420	ASP	C	425	LEU
A	415	THR	B	421	TYR	C	426	PRO
A	416	GLY	B	422	ASN	C	427	ASP
A	417	LYS	B	424	LYS	C	428	ASP
A	418	ILE	B	425	LEU	C	429	PHE
A	420	ASP	B	426	PRO	C	430	THR
A	421	TYR	B	427	ASP	C	433	VAL
A	422	ASN	B	428	ASP	C	435	ALA
A	424	LYS	B	429	PHE	C	436	TRP
A	425	LEU	B	433	VAL	C	437	ASN
A	426	PRO	B	435	ALA	C	439	ASN
A	427	ASP	B	436	TRP	C	440	ASN
A	428	ASP	B	437	ASN	C	441	LEU
A	429	PHE	B	439	ASN	C	443	SER
A	430	THR	B	440	ASN	C	449	TYR

A	337	PRO	B	348	ALA	C	323	THR
A	338	PHE	B	349	SER	C	324	GLU
A	339	GLY	B	351	TYR	C	325	SER
A	340	GLU	B	352	ALA	C	327	VAL
A	341	VAL	B	353	TRP	C	330	PRO
A	342	PHE	B	354	ASN	C	331	ASN
A	343	ASN	B	355	ARG	C	332	ILE
A	344	ALA	B	356	LYS	C	333	THR
A	345	THR	B	357	ARG	C	334	ASN
A	346	ARG	B	359	SER	C	335	LEU
A	347	PHE	B	360	ASN	C	336	CYS
A	348	ALA	B	361	CYS	C	337	PRO
A	349	SER	B	364	ASP	C	339	GLY
A	351	TYR	B	366	SER	C	340	GLU
A	352	ALA	B	367	VAL	C	341	VAL
A	353	TRP	B	369	TYR	C	342	PHE
A	354	ASN	B	370	ASN	C	343	ASN
A	355	ARG	B	371	SER	C	344	ALA
A	356	LYS	B	372	ALA	C	345	THR
A	357	ARG	B	373	SER	C	346	ARG
A	358	ILE	B	374	PHE	C	347	PHE
A	359	SER	B	375	SER	C	349	SER
A	360	ASN	B	376	THR	C	352	ALA
A	362	VAL	B	377	PHE	C	354	ASN
A	366	SER	B	378	LYS	C	355	ARG
A	367	VAL	B	379	CYS	C	356	LYS
A	368	LEU	B	380	TYR	C	357	ARG
A	369	TYR	B	381	GLY	C	359	SER
A	370	ASN	B	382	VAL	C	360	ASN
A	371	SER	B	383	SER	C	362	VAL
A	372	ALA	B	384	PRO	C	364	ASP
A	373	SER	B	385	THR	C	366	SER
A	374	PHE	B	386	LYS	C	367	VAL
A	375	SER	B	388	ASN	C	369	TYR
A	376	THR	B	389	ASP	C	370	ASN
A	377	PHE	B	390	LEU	C	371	SER
A	378	LYS	B	391	CYS	C	372	ALA
A	379	CYS	B	392	PHE	C	373	SER
A	380	TYR	B	393	THR	C	374	PHE
A	384	PRO	B	394	ASN	C	375	SER
A	385	THR	B	399	SER	C	376	THR
A	386	LYS	B	402	ILE	C	377	PHE
A	388	ASN	B	403	ARG	C	378	LYS
A	389	ASP	B	404	GLY	C	379	CYS
A	390	LEU	B	405	ASP	C	380	TYR
A	393	THR	B	406	GLU	C	381	GLY
A	394	ASN	B	407	VAL	C	382	VAL
A	396	TYR	B	408	ARG	C	384	PRO
A	397	ALA	B	409	GLN	C	385	THR
A	399	SER	B	411	ALA	C	386	LYS
A	403	ARG	B	412	PRO	C	388	ASN
A	404	GLY	B	413	GLY	C	389	ASP
A	405	ASP	B	414	GLN	C	393	THR
A	406	GLU	B	415	THR	C	394	ASN
A	407	VAL	B	416	GLY	C	399	SER
A	408	ARG	B	417	LYS	C	404	GLY
A	409	GLN	B	418	ILE	C	405	ASP
A	410	ILE	B	419	ALA	C	407	VAL
A	411	ALA	B	420	ASP	C	408	ARG
A	412	PRO	B	421	TYR	C	409	GLN
A	413	GLY	B	422	ASN	C	411	ALA
A	414	GLN	B	424	LYS	C	412	PRO
A	415	THR	B	425	LEU	C	413	GLY
A	416	GLY	B	426	PRO	C	414	GLN
A	417	LYS	B	427	ASP	C	415	THR
A	418	ILE	B	428	ASP	C	416	GLY
A	420	ASP	B	429	PHE	C	417	LYS
A	421	TYR	B	433	VAL	C	420	ASP
A	422	ASN	B	435	ALA	C	421	TYR
A	424	LYS	B	436	TRP	C	426	PRO
A	426	PRO	B	437	ASN	C	427	ASP
A	427	ASP	B	439	ASN	C	428	ASP
A	428	ASP	B	440	ASN	C	429	PHE
A	429	PHE	B	441	LEU	C	430	THR
A	430	THR	B	443	SER	C	433	VAL
A	433	VAL	B	449	TYR	C	436	TRP
A	435	ALA	B	450	ASN	C	437	ASN
A	436	TRP	B	451	TYR	C	439	ASN
A	437	ASN	B	452	LEU	C	440	ASN
A	438	SER	B	453	TYR	C	441	LEU
A	439	ASN	B	454	ARG	C	443	SER
A	440	ASN	B	460	ASN	C	449	TYR
A	441	LEU	B	461	LEU	C	450	ASN
A	442	ASP	B	462	LYS	C	451	TYR
A	443	SER	B	463	PRO	C	452	LEU
A	449	TYR	B	464	PHE	C	454	ARG
A	450	ASN	B	465	GLU	C	460	ASN
A	451	TYR	B	466	ARG	C	462	LYS
A	452	LEU	B	467	ASP	C	463	PRO
A	453	TYR	B	468	ILE	C	466	ARG

A	387	LEU	B	388	ASN	C	372	ALA
A	388	ASN	B	389	ASP	C	373	SER
A	389	ASP	B	390	LEU	C	374	PHE
A	390	LEU	B	391	CYS	C	375	SER
A	391	CYS	B	392	PHE	C	376	THR
A	392	PHE	B	393	THR	C	377	PHE
A	394	ASN	B	394	ASN	C	378	LYS
A	396	TYR	B	396	TYR	C	379	CYS
A	397	ALA	B	399	SER	C	380	TYR
A	399	SER	B	402	ILE	C	381	GLY
A	403	ARG	B	403	ARG	C	382	VAL
A	404	GLY	B	404	GLY	C	384	PRO
A	405	ASP	B	405	ASP	C	385	THR
A	406	GLU	B	406	GLU	C	386	LYS
A	407	VAL	B	407	VAL	C	388	ASN
A	408	ARG	B	408	ARG	C	389	ASP
A	409	GLN	B	409	GLN	C	390	LEU
A	411	ALA	B	411	ALA	C	391	CYS
A	412	PRO	B	412	PRO	C	393	THR
A	413	GLY	B	413	GLY	C	394	ASN
A	414	GLN	B	414	GLN	C	396	TYR
A	415	THR	B	415	THR	C	399	SER
A	416	GLY	B	416	GLY	C	401	VAL
A	417	LYS	B	417	LYS	C	402	ILE
A	418	ILE	B	418	ILE	C	403	ARG
A	419	ALA	B	419	ALA	C	404	GLY
A	420	ASP	B	420	ASP	C	405	ASP
A	421	TYR	B	421	TYR	C	406	GLU
A	422	ASN	B	422	ASN	C	407	VAL
A	423	TYR	B	424	LYS	C	408	ARG
A	424	LYS	B	425	LEU	C	409	GLN
A	425	LEU	B	426	PRO	C	410	ILE
A	426	PRO	B	427	ASP	C	411	ALA
A	427	ASP	B	428	ASP	C	412	PRO
A	428	ASP	B	429	PHE	C	413	GLY
A	429	PHE	B	433	VAL	C	414	GLN
A	430	THR	B	435	ALA	C	415	THR
A	432	CYS	B	436	TRP	C	416	GLY
A	433	VAL	B	437	ASN	C	417	LYS
A	435	ALA	B	438	SER	C	418	ILE
A	436	TRP	B	439	ASN	C	419	ALA
A	437	ASN	B	440	ASN	C	420	ASP
A	438	SER	B	441	LEU	C	421	TYR
A	439	ASN	B	442	ASP	C	422	ASN
A	440	ASN	B	443	SER	C	424	LYS
A	441	LEU	B	449	TYR	C	425	LEU
A	442	ASP	B	450	ASN	C	426	PRO
A	443	SER	B	451	TYR	C	427	ASP
A	449	TYR	B	452	LEU	C	428	ASP
A	450	ASN	B	453	TYR	C	429	PHE
A	451	TYR	B	454	ARG	C	433	VAL
A	452	LEU	B	460	ASN	C	435	ALA
A	453	TYR	B	461	LEU	C	436	TRP
A	454	ARG	B	462	LYS	C	437	ASN
A	491	PRO	B	463	PRO	C	438	SER
A	492	LEU	B	464	PHE	C	439	ASN
A	493	GLN	B	465	GLU	C	440	ASN
A	494	SER	B	466	ARG	C	441	LEU
A	495	TYR	B	467	ASP	C	442	ASP
A	496	GLY	B	468	ILE	C	443	SER
A	497	PHE	B	469	SER	C	449	TYR
A	498	GLN	B	470	THR	C	450	ASN
A	499	PRO	B	471	GLU	C	451	TYR
A	500	THR	B	472	ILE	C	452	LEU
A	503	VAL	B	473	TYR	C	453	TYR
A	504	GLY	B	487	ASN	C	454	ARG
A	505	TYR	B	488	CYS	C	460	ASN
A	506	GLN	B	489	TYR	C	461	LEU
A	508	TYR	B	490	PHE	C	462	LYS
A	509	ARG	B	491	PRO	C	463	PRO
A	512	VAL	B	492	LEU	C	464	PHE
A	513	LEU	B	493	GLN	C	465	GLU
A	514	SER	B	494	SER	C	466	ARG
A	515	PHE	B	495	TYR	C	467	ASP
A	516	GLU	B	496	GLY	C	468	ILE
A	517	LEU	B	497	PHE	C	469	SER
A	518	LEU	B	498	GLN	C	470	THR
A	519	HIS	B	499	PRO	C	471	GLU
A	520	ALA	B	500	THR	C	487	ASN
A	521	PRO	B	503	VAL	C	488	CYS
A	522	ALA	B	504	GLY	C	489	TYR
A	523	THR	B	505	TYR	C	490	PHE
A	525	CYS	B	506	GLN	C	491	PRO
A	526	GLY	B	507	PRO	C	492	LEU
A	527	PRO	B	508	TYR	C	493	GLN
A	528	LYS	B	509	ARG	C	494	SER
A	529	LYS	B	510	VAL	C	495	TYR
A	530	SER	B	513	LEU	C	496	GLY
A	531	THR	B	514	SER	C	497	PHE
A	532	ASN	B	515	PHE	C	498	GLN

A	432	CYS	B	441	LEU	C	450	ASN
A	433	VAL	B	443	SER	C	451	TYR
A	435	ALA	B	449	TYR	C	452	LEU
A	436	TRP	B	450	ASN	C	453	TYR
A	437	ASN	B	451	TYR	C	454	ARG
A	438	SER	B	452	LEU	C	460	ASN
A	439	ASN	B	453	TYR	C	462	LYS
A	440	ASN	B	454	ARG	C	463	PRO
A	441	LEU	B	460	ASN	C	466	ARG
A	442	ASP	B	461	LEU	C	468	ILE
A	443	SER	B	462	LYS	C	469	SER
A	449	TYR	B	463	PRO	C	470	THR
A	450	ASN	B	464	PHE	C	471	GLU
A	451	TYR	B	465	GLU	C	487	ASN
A	452	LEU	B	466	ARG	C	488	CYS
A	453	TYR	B	467	ASP	C	489	TYR
A	454	ARG	B	468	ILE	C	490	PHE
A	491	PRO	B	469	SER	C	491	PRO
A	492	LEU	B	470	THR	C	492	LEU
A	493	GLN	B	471	GLU	C	493	GLN
A	494	SER	B	472	ILE	C	494	SER
A	495	TYR	B	473	TYR	C	496	GLY
A	496	GLY	B	487	ASN	C	498	GLN
A	497	PHE	B	488	CYS	C	503	VAL
A	498	GLN	B	489	TYR	C	504	GLY
A	499	PRO	B	490	PHE	C	505	TYR
A	500	THR	B	491	PRO	C	506	GLN
A	503	VAL	B	492	LEU	C	508	TYR
A	504	GLY	B	493	GLN	C	514	SER
A	505	TYR	B	494	SER	C	515	PHE
A	506	GLN	B	495	TYR	C	516	GLU
A	508	TYR	B	496	GLY	C	517	LEU
A	509	ARG	B	497	PHE	C	518	LEU
A	514	SER	B	498	GLN	C	519	HIS
A	515	PHE	B	499	PRO	C	520	ALA
A	516	GLU	B	500	THR	C	521	PRO
A	517	LEU	B	503	VAL	C	522	ALA
A	518	LEU	B	504	GLY	C	523	THR
A	519	HIS	B	505	TYR	C	527	PRO
A	520	ALA	B	506	GLN	C	528	LYS
A	521	PRO	B	507	PRO	C	529	LYS
A	522	ALA	B	508	TYR	C	530	SER
A	523	THR	B	509	ARG	C	531	THR
A	527	PRO	B	510	VAL	C	532	ASN
A	528	LYS	B	514	SER	C	533	LEU
A	529	LYS	B	515	PHE	C	534	VAL
A	530	SER	B	516	GLU	C	535	LYS
A	531	THR	B	517	LEU	C	536	ASN
A	532	ASN	B	518	LEU	C	537	LYS
A	533	LEU	B	519	HIS	C	538	CYS
A	534	VAL	B	520	ALA	C	540	ASN
A	535	LYS	B	521	PRO	C	542	ASN
A	536	ASN	B	522	ALA	C	544	ASN
A	537	LYS	B	523	THR	C	545	GLY
A	538	CYS	B	527	PRO	C	547	THR
A	540	ASN	B	528	LYS	C	548	GLY
A	541	PHE	B	529	LYS	C	549	THR
A	542	ASN	B	530	SER	C	551	VAL
A	545	GLY	B	531	THR	C	553	THR
A	546	LEU	B	532	ASN	C	554	GLU
A	547	THR	B	533	LEU	C	555	SER
A	548	GLY	B	534	VAL	C	556	ASN
A	549	THR	B	535	LYS	C	557	LYS
A	551	VAL	B	536	ASN	C	558	LYS
A	553	THR	B	537	LYS	C	559	PHE
A	554	GLU	B	538	CYS	C	560	LEU
A	555	SER	B	540	ASN	C	561	PRO
A	556	ASN	B	541	PHE	C	562	PHE
A	557	LYS	B	542	ASN	C	564	GLN
A	558	LYS	B	544	ASN	C	567	ARG
A	559	PHE	B	545	GLY	C	568	ASP
A	560	LEU	B	546	LEU	C	569	ILE
A	561	PRO	B	547	THR	C	570	ALA
A	562	PHE	B	548	GLY	C	571	ASP
A	564	GLN	B	549	THR	C	572	THR
A	567	ARG	B	551	VAL	C	573	THR
A	568	ASP	B	553	THR	C	574	ASP
A	569	ILE	B	554	GLU	C	577	ARG
A	570	ALA	B	555	SER	C	579	PRO
A	571	ASP	B	556	ASN	C	580	GLN
A	572	THR	B	557	LYS	C	581	THR
A	573	THR	B	558	LYS	C	582	LEU
A	574	ASP	B	559	PHE	C	583	GLU
A	577	ARG	B	560	LEU	C	584	ILE
A	579	PRO	B	561	PRO	C	586	ASP
A	580	GLN	B	562	PHE	C	587	ILE
A	581	THR	B	564	GLN	C	588	THR
A	582	LEU	B	565	PHE	C	589	PRO
A	583	GLU	B	567	ARG	C	590	CYS
A	584	ILE	B	568	ASP	C	591	SER

A	454	ARG	B	469	SER	C	468	ILE
A	491	PRO	B	470	THR	C	469	SER
A	492	LEU	B	471	GLU	C	470	THR
A	493	GLN	B	472	ILE	C	471	GLU
A	494	SER	B	473	TYR	C	487	ASN
A	495	TYR	B	487	ASN	C	488	CYS
A	496	GLY	B	488	CYS	C	489	TYR
A	497	PHE	B	489	TYR	C	490	PHE
A	498	GLN	B	490	PHE	C	491	PRO
A	499	PRO	B	491	PRO	C	492	LEU
A	500	THR	B	492	LEU	C	493	GLN
A	503	VAL	B	493	GLN	C	494	SER
A	504	GLY	B	494	SER	C	496	GLY
A	505	TYR	B	495	TYR	C	498	GLN
A	506	GLN	B	496	GLY	C	503	VAL
A	508	TYR	B	497	PHE	C	504	GLY
A	509	ARG	B	498	GLN	C	505	TYR
A	514	SER	B	499	PRO	C	506	GLN
A	515	PHE	B	500	THR	C	508	TYR
A	516	GLU	B	503	VAL	C	514	SER
A	517	LEU	B	504	GLY	C	515	PHE
A	518	LEU	B	505	TYR	C	517	LEU
A	521	PRO	B	506	GLN	C	518	LEU
A	522	ALA	B	508	TYR	C	519	HIS
A	523	THR	B	509	ARG	C	521	PRO
A	527	PRO	B	510	VAL	C	522	ALA
A	528	LYS	B	514	SER	C	523	THR
A	529	LYS	B	515	PHE	C	527	PRO
A	530	SER	B	516	GLU	C	528	LYS
A	531	THR	B	517	LEU	C	529	LYS
A	532	ASN	B	518	LEU	C	530	SER
A	533	LEU	B	519	HIS	C	531	THR
A	534	VAL	B	520	ALA	C	532	ASN
A	535	LYS	B	521	PRO	C	533	LEU
A	536	ASN	B	522	ALA	C	534	VAL
A	537	LYS	B	523	THR	C	535	LYS
A	538	CYS	B	527	PRO	C	536	ASN
A	540	ASN	B	528	LYS	C	537	LYS
A	542	ASN	B	529	LYS	C	538	CYS
A	545	GLY	B	530	SER	C	540	ASN
A	547	THR	B	531	THR	C	542	ASN
A	548	GLY	B	532	ASN	C	544	ASN
A	549	THR	B	533	LEU	C	547	THR
A	551	VAL	B	534	VAL	C	548	GLY
A	553	THR	B	535	LYS	C	549	THR
A	554	GLU	B	536	ASN	C	551	VAL
A	555	SER	B	537	LYS	C	553	THR
A	556	ASN	B	538	CYS	C	554	GLU
A	557	LYS	B	540	ASN	C	555	SER
A	558	LYS	B	542	ASN	C	556	ASN
A	559	PHE	B	544	ASN	C	557	LYS
A	560	LEU	B	545	GLY	C	558	LYS
A	561	PRO	B	546	LEU	C	559	PHE
A	562	PHE	B	547	THR	C	560	LEU
A	567	ARG	B	548	GLY	C	561	PRO
A	568	ASP	B	549	THR	C	562	PHE
A	569	ILE	B	551	VAL	C	564	GLN
A	570	ALA	B	553	THR	C	567	ARG
A	571	ASP	B	554	GLU	C	568	ASP
A	572	THR	B	555	SER	C	569	ILE
A	574	ASP	B	556	ASN	C	571	ASP
A	577	ARG	B	557	LYS	C	572	THR
A	579	PRO	B	558	LYS	C	573	THR
A	580	GLN	B	559	PHE	C	574	ASP
A	581	THR	B	560	LEU	C	577	ARG
A	582	LEU	B	561	PRO	C	579	PRO
A	583	GLU	B	562	PHE	C	580	GLN
A	584	ILE	B	564	GLN	C	581	THR
A	586	ASP	B	565	PHE	C	582	LEU
A	587	ILE	B	567	ARG	C	583	GLU
A	588	THR	B	568	ASP	C	584	ILE
A	589	PRO	B	569	ILE	C	586	ASP
A	591	SER	B	570	ALA	C	588	THR
A	594	GLY	B	571	ASP	C	589	PRO
A	601	GLY	B	572	THR	C	590	CYS
A	602	THR	B	573	THR	C	591	SER
A	603	ASN	B	574	ASP	C	592	PHE
A	604	THR	B	577	ARG	C	595	VAL
A	605	SER	B	580	GLN	C	596	SER
A	606	ASN	B	581	THR	C	600	PRO
A	607	GLN	B	582	LEU	C	602	THR
A	608	VAL	B	583	GLU	C	603	ASN
A	610	VAL	B	584	ILE	C	604	THR
A	612	TYR	B	586	ASP	C	605	SER
A	614	ASP	B	587	ILE	C	606	ASN
A	615	VAL	B	588	THR	C	607	GLN
A	616	ASN	B	589	PRO	C	608	VAL
A	617	CYS	B	590	CYS	C	610	VAL
A	618	THR	B	591	SER	C	612	TYR
A	619	GLU	B	592	PHE	C	616	ASN

A	533	LEU	B	516	GLU	C	503	VAL
A	534	VAL	B	517	LEU	C	504	GLY
A	535	LYS	B	518	LEU	C	505	TYR
A	536	ASN	B	519	HIS	C	506	GLN
A	537	LYS	B	520	ALA	C	508	TYR
A	538	CYS	B	521	PRO	C	509	ARG
A	540	ASN	B	522	ALA	C	513	LEU
A	541	PHE	B	523	THR	C	514	SER
A	542	ASN	B	525	CYS	C	515	PHE
A	543	PHE	B	526	GLY	C	516	GLU
A	544	ASN	B	527	PRO	C	517	LEU
A	545	GLY	B	528	LYS	C	518	LEU
A	546	LEU	B	529	LYS	C	519	HIS
A	547	THR	B	530	SER	C	520	ALA
A	548	GLY	B	531	THR	C	521	PRO
A	549	THR	B	532	ASN	C	522	ALA
A	551	VAL	B	533	LEU	C	523	THR
A	552	LEU	B	534	VAL	C	525	CYS
A	553	THR	B	535	LYS	C	527	PRO
A	554	GLU	B	536	ASN	C	528	LYS
A	555	SER	B	537	LYS	C	529	LYS
A	556	ASN	B	538	CYS	C	530	SER
A	557	LYS	B	540	ASN	C	531	THR
A	558	LYS	B	541	PHE	C	532	ASN
A	559	PHE	B	542	ASN	C	533	LEU
A	560	LEU	B	543	PHE	C	534	VAL
A	561	PRO	B	544	ASN	C	535	LYS
A	562	PHE	B	545	GLY	C	536	ASN
A	564	GLN	B	546	LEU	C	537	LYS
A	565	PHE	B	547	THR	C	538	CYS
A	567	ARG	B	548	GLY	C	539	VAL
A	568	ASP	B	549	THR	C	540	ASN
A	569	ILE	B	551	VAL	C	541	PHE
A	570	ALA	B	552	LEU	C	542	ASN
A	571	ASP	B	553	THR	C	543	PHE
A	572	THR	B	554	GLU	C	544	ASN
A	573	THR	B	555	SER	C	545	GLY
A	574	ASP	B	556	ASN	C	546	LEU
A	576	VAL	B	557	LYS	C	547	THR
A	577	ARG	B	558	LYS	C	548	GLY
A	579	PRO	B	559	PHE	C	549	THR
A	580	GLN	B	560	LEU	C	551	VAL
A	581	THR	B	561	PRO	C	552	LEU
A	582	LEU	B	562	PHE	C	553	THR
A	583	GLU	B	563	GLN	C	554	GLU
A	584	ILE	B	564	GLN	C	555	SER
A	585	LEU	B	565	PHE	C	556	ASN
A	586	ASP	B	567	ARG	C	557	LYS
A	587	ILE	B	568	ASP	C	558	LYS
A	588	THR	B	569	ILE	C	559	PHE
A	589	PRO	B	570	ALA	C	560	LEU
A	590	CYS	B	571	ASP	C	561	PRO
A	591	SER	B	572	THR	C	562	PHE
A	592	PHE	B	573	THR	C	563	GLN
A	593	GLY	B	574	ASP	C	564	GLN
A	594	GLY	B	576	VAL	C	565	PHE
A	595	VAL	B	577	ARG	C	567	ARG
A	596	SER	B	578	ASP	C	568	ASP
A	600	PRO	B	579	PRO	C	569	ILE
A	601	GLY	B	580	GLN	C	570	ALA
A	602	THR	B	581	THR	C	571	ASP
A	603	ASN	B	582	LEU	C	572	THR
A	604	THR	B	583	GLU	C	573	THR
A	605	SER	B	584	ILE	C	574	ASP
A	606	ASN	B	585	LEU	C	576	VAL
A	607	GLN	B	586	ASP	C	577	ARG
A	608	VAL	B	587	ILE	C	578	ASP
A	610	VAL	B	588	THR	C	579	PRO
A	611	LEU	B	589	PRO	C	580	GLN
A	612	TYR	B	590	CYS	C	581	THR
A	613	GLN	B	591	SER	C	582	LEU
A	614	ASP	B	592	PHE	C	583	GLU
A	615	VAL	B	593	GLY	C	584	ILE
A	616	ASN	B	594	GLY	C	586	ASP
A	617	CYS	B	595	VAL	C	587	ILE
A	618	THR	B	596	SER	C	588	THR
A	619	GLU	B	597	VAL	C	589	PRO
A	620	VAL	B	599	THR	C	590	CYS
A	640	SER	B	600	PRO	C	591	SER
A	641	ASN	B	601	GLY	C	592	PHE
A	642	VAL	B	602	THR	C	593	GLY
A	643	PHE	B	603	ASN	C	594	GLY
A	644	GLN	B	604	THR	C	595	VAL
A	645	THR	B	605	SER	C	596	SER
A	646	ARG	B	606	ASN	C	597	VAL
A	647	ALA	B	607	GLN	C	600	PRO
A	649	CYS	B	608	VAL	C	601	GLY
A	651	ILE	B	610	VAL	C	602	THR
A	652	GLY	B	611	LEU	C	603	ASN
A	654	GLU	B	612	TYR	C	604	THR

A	586	ASP	B	569	ILE	C	592	PHE
A	587	ILE	B	570	ALA	C	595	VAL
A	588	THR	B	571	ASP	C	596	SER
A	589	PRO	B	572	THR	C	600	PRO
A	591	SER	B	573	THR	C	602	THR
A	594	GLY	B	574	ASP	C	603	ASN
A	602	THR	B	577	ARG	C	604	THR
A	603	ASN	B	578	ASP	C	605	SER
A	604	THR	B	580	GLN	C	606	ASN
A	605	SER	B	581	THR	C	607	GLN
A	606	ASN	B	582	LEU	C	608	VAL
A	607	GLN	B	583	GLU	C	610	VAL
A	608	VAL	B	584	ILE	C	612	TYR
A	610	VAL	B	586	ASP	C	613	GLN
A	611	LEU	B	587	ILE	C	616	ASN
A	612	TYR	B	588	THR	C	617	CYS
A	613	GLN	B	589	PRO	C	618	THR
A	614	ASP	B	590	CYS	C	619	GLU
A	615	VAL	B	591	SER	C	620	VAL
A	616	ASN	B	592	PHE	C	641	ASN
A	617	CYS	B	593	GLY	C	642	VAL
A	618	THR	B	596	SER	C	643	PHE
A	619	GLU	B	600	PRO	C	644	GLN
A	620	VAL	B	602	THR	C	645	THR
A	640	SER	B	603	ASN	C	646	ARG
A	641	ASN	B	604	THR	C	651	ILE
A	642	VAL	B	605	SER	C	652	GLY
A	643	PHE	B	606	ASN	C	654	GLU
A	644	GLN	B	607	GLN	C	655	HIS
A	646	ARG	B	608	VAL	C	656	VAL
A	651	ILE	B	612	TYR	C	657	ASN
A	652	GLY	B	613	GLN	C	658	ASN
A	654	GLU	B	614	ASP	C	659	SER
A	655	HIS	B	615	VAL	C	660	TYR
A	656	VAL	B	616	ASN	C	661	GLU
A	657	ASN	B	617	CYS	C	662	CYS
A	658	ASN	B	618	THR	C	663	ASP
A	659	SER	B	619	GLU	C	665	PRO
A	660	TYR	B	620	VAL	C	666	ILE
A	661	GLU	B	638	THR	C	668	ALA
A	662	CYS	B	639	GLY	C	670	ILE
A	663	ASP	B	640	SER	C	687	VAL
A	665	PRO	B	641	ASN	C	688	ALA
A	666	ILE	B	642	VAL	C	689	SER
A	668	ALA	B	643	PHE	C	690	GLN
A	670	ILE	B	644	GLN	C	691	SER
A	687	VAL	B	645	THR	C	696	THR
A	688	ALA	B	646	ARG	C	697	MET
A	690	GLN	B	651	ILE	C	698	SER
A	691	SER	B	652	GLY	C	699	LEU
A	693	ILE	B	654	GLU	C	700	GLY
A	696	THR	B	655	HIS	C	701	ALA
A	697	MET	B	656	VAL	C	702	GLU
A	698	SER	B	657	ASN	C	703	ASN
A	699	LEU	B	658	ASN	C	704	SER
A	700	GLY	B	659	SER	C	706	ALA
A	701	ALA	B	660	TYR	C	707	TYR
A	702	GLU	B	661	GLU	C	708	SER
A	703	ASN	B	662	CYS	C	709	ASN
A	704	SER	B	663	ASP	C	710	ASN
A	705	VAL	B	665	PRO	C	711	SER
A	706	ALA	B	666	ILE	C	712	ILE
A	707	TYR	B	668	ALA	C	717	ASN
A	708	SER	B	670	ILE	C	719	THR
A	709	ASN	B	688	ALA	C	720	ILE
A	710	ASN	B	689	SER	C	721	SER
A	711	SER	B	690	GLN	C	722	VAL
A	712	ILE	B	691	SER	C	723	THR
A	716	THR	B	693	ILE	C	724	THR
A	717	ASN	B	696	THR	C	725	GLU
A	719	THR	B	697	MET	C	726	ILE
A	720	ILE	B	698	SER	C	727	LEU
A	721	SER	B	699	LEU	C	728	PRO
A	722	VAL	B	700	GLY	C	730	SER
A	723	THR	B	701	ALA	C	732	THR
A	724	THR	B	702	GLU	C	733	LYS
A	725	GLU	B	703	ASN	C	739	THR
A	726	ILE	B	704	SER	C	740	MET
A	727	LEU	B	705	VAL	C	743	CYS
A	728	PRO	B	706	ALA	C	744	GLY
A	730	SER	B	707	TYR	C	745	ASP
A	732	THR	B	708	SER	C	747	THR
A	733	LYS	B	709	ASN	C	748	GLU
A	737	ASP	B	710	ASN	C	750	SER
A	739	THR	B	711	SER	C	751	ASN
A	740	MET	B	713	ALA	C	752	LEU
A	744	GLY	B	717	ASN	C	754	LEU
A	745	ASP	B	719	THR	C	755	GLN
A	747	THR	B	720	ILE	C	756	TYR
A	748	GLU	B	721	SER	C	757	GLY

A	620	VAL	B	600	PRO	C	617	CYS
A	640	SER	B	602	THR	C	618	THR
A	641	ASN	B	603	ASN	C	619	GLU
A	642	VAL	B	604	THR	C	620	VAL
A	643	PHE	B	605	SER	C	641	ASN
A	644	GLN	B	606	ASN	C	642	VAL
A	646	ARG	B	607	GLN	C	643	PHE
A	651	ILE	B	608	VAL	C	644	GLN
A	652	GLY	B	612	TYR	C	645	THR
A	654	GLU	B	613	GLN	C	646	ARG
A	655	HIS	B	614	ASP	C	651	ILE
A	656	VAL	B	615	VAL	C	652	GLY
A	657	ASN	B	616	ASN	C	654	GLU
A	658	ASN	B	617	CYS	C	655	HIS
A	659	SER	B	618	THR	C	656	VAL
A	660	TYR	B	619	GLU	C	657	ASN
A	661	GLU	B	620	VAL	C	658	ASN
A	662	CYS	B	638	THR	C	659	SER
A	663	ASP	B	639	GLY	C	660	TYR
A	665	PRO	B	640	SER	C	661	GLU
A	666	ILE	B	641	ASN	C	662	CYS
A	668	ALA	B	642	VAL	C	663	ASP
A	670	ILE	B	643	PHE	C	665	PRO
A	687	VAL	B	644	GLN	C	666	ILE
A	690	GLN	B	645	THR	C	668	ALA
A	691	SER	B	646	ARG	C	670	ILE
A	693	ILE	B	651	ILE	C	687	VAL
A	696	THR	B	652	GLY	C	688	ALA
A	697	MET	B	654	GLU	C	690	GLN
A	698	SER	B	655	HIS	C	691	SER
A	699	LEU	B	656	VAL	C	696	THR
A	700	GLY	B	657	ASN	C	697	MET
A	701	ALA	B	658	ASN	C	698	SER
A	702	GLU	B	659	SER	C	699	LEU
A	703	ASN	B	660	TYR	C	700	GLY
A	704	SER	B	661	GLU	C	701	ALA
A	706	ALA	B	662	CYS	C	702	GLU
A	707	TYR	B	663	ASP	C	703	ASN
A	708	SER	B	665	PRO	C	704	SER
A	709	ASN	B	666	ILE	C	706	ALA
A	710	ASN	B	668	ALA	C	707	TYR
A	711	SER	B	670	ILE	C	708	SER
A	712	ILE	B	688	ALA	C	709	ASN
A	716	THR	B	689	SER	C	710	ASN
A	717	ASN	B	690	GLN	C	711	SER
A	719	THR	B	691	SER	C	716	THR
A	720	ILE	B	693	ILE	C	717	ASN
A	721	SER	B	696	THR	C	719	THR
A	722	VAL	B	697	MET	C	720	ILE
A	723	THR	B	698	SER	C	721	SER
A	724	THR	B	699	LEU	C	722	VAL
A	725	GLU	B	700	GLY	C	723	THR
A	727	LEU	B	701	ALA	C	724	THR
A	728	PRO	B	702	GLU	C	725	GLU
A	730	SER	B	703	ASN	C	726	ILE
A	732	THR	B	704	SER	C	727	LEU
A	733	LYS	B	705	VAL	C	728	PRO
A	737	ASP	B	706	ALA	C	730	SER
A	739	THR	B	707	TYR	C	732	THR
A	740	MET	B	708	SER	C	733	LYS
A	744	GLY	B	709	ASN	C	739	THR
A	745	ASP	B	710	ASN	C	740	MET
A	747	THR	B	711	SER	C	743	CYS
A	748	GLU	B	713	ALA	C	744	GLY
A	750	SER	B	716	THR	C	745	ASP
A	751	ASN	B	717	ASN	C	747	THR
A	754	LEU	B	719	THR	C	748	GLU
A	755	GLN	B	720	ILE	C	750	SER
A	757	GLY	B	721	SER	C	751	ASN
A	758	SER	B	722	VAL	C	754	LEU
A	760	CYS	B	723	THR	C	755	GLN
A	761	THR	B	724	THR	C	757	GLY
A	762	GLN	B	725	GLU	C	758	SER
A	764	ASN	B	726	ILE	C	760	CYS
A	765	ARG	B	727	LEU	C	761	THR
A	766	ALA	B	728	PRO	C	764	ASN
A	768	THR	B	730	SER	C	765	ARG
A	769	GLY	B	732	THR	C	766	ALA
A	772	VAL	B	733	LYS	C	768	THR
A	773	GLU	B	737	ASP	C	769	GLY
A	775	ASP	B	739	THR	C	770	ILE
A	776	LYS	B	740	MET	C	772	VAL
A	778	THR	B	744	GLY	C	773	GLU
A	779	GLN	B	745	ASP	C	775	ASP
A	780	GLU	B	747	THR	C	776	LYS
A	783	ALA	B	748	GLU	C	778	THR
A	784	GLN	B	750	SER	C	779	GLN
A	785	VAL	B	751	ASN	C	780	GLU
A	786	LYS	B	752	LEU	C	783	ALA
A	787	GLN	B	753	LEU	C	784	GLN

A	655	HIS	B	613	GLN	C	605	SER
A	656	VAL	B	614	ASP	C	606	ASN
A	657	ASN	B	615	VAL	C	607	GLN
A	658	ASN	B	616	ASN	C	608	VAL
A	659	SER	B	617	CYS	C	610	VAL
A	660	TYR	B	618	THR	C	611	LEU
A	661	GLU	B	619	GLU	C	612	TYR
A	662	CYS	B	620	VAL	C	613	GLN
A	663	ASP	B	638	THR	C	614	ASP
A	664	ILE	B	639	GLY	C	615	VAL
A	665	PRO	B	640	SER	C	616	ASN
A	666	ILE	B	641	ASN	C	617	CYS
A	668	ALA	B	642	VAL	C	618	THR
A	670	ILE	B	643	PHE	C	619	GLU
A	672	ALA	B	644	GLN	C	620	VAL
A	687	VAL	B	645	THR	C	641	ASN
A	688	ALA	B	646	ARG	C	642	VAL
A	689	SER	B	647	ALA	C	643	PHE
A	690	GLN	B	650	LEU	C	644	GLN
A	691	SER	B	651	ILE	C	645	THR
A	693	ILE	B	652	GLY	C	646	ARG
A	695	TYR	B	654	GLU	C	647	ALA
A	696	THR	B	655	HIS	C	649	CYS
A	697	MET	B	656	VAL	C	651	ILE
A	698	SER	B	657	ASN	C	652	GLY
A	699	LEU	B	658	ASN	C	654	GLU
A	700	GLY	B	659	SER	C	655	HIS
A	701	ALA	B	660	TYR	C	656	VAL
A	702	GLU	B	661	GLU	C	657	ASN
A	703	ASN	B	662	CYS	C	658	ASN
A	704	SER	B	663	ASP	C	659	SER
A	705	VAL	B	664	ILE	C	660	TYR
A	706	ALA	B	665	PRO	C	661	GLU
A	707	TYR	B	666	ILE	C	662	CYS
A	708	SER	B	668	ALA	C	663	ASP
A	709	ASN	B	670	ILE	C	664	ILE
A	710	ASN	B	672	ALA	C	665	PRO
A	711	SER	B	687	VAL	C	666	ILE
A	712	ILE	B	688	ALA	C	668	ALA
A	713	ALA	B	689	SER	C	670	ILE
A	716	THR	B	690	GLN	C	687	VAL
A	717	ASN	B	691	SER	C	688	ALA
A	719	THR	B	693	ILE	C	689	SER
A	720	ILE	B	695	TYR	C	690	GLN
A	721	SER	B	696	THR	C	691	SER
A	722	VAL	B	697	MET	C	693	ILE
A	723	THR	B	698	SER	C	695	TYR
A	724	THR	B	699	LEU	C	696	THR
A	725	GLU	B	700	GLY	C	697	MET
A	726	ILE	B	701	ALA	C	698	SER
A	727	LEU	B	702	GLU	C	699	LEU
A	728	PRO	B	703	ASN	C	700	GLY
A	730	SER	B	704	SER	C	701	ALA
A	731	MET	B	705	VAL	C	702	GLU
A	732	THR	B	706	ALA	C	703	ASN
A	733	LYS	B	707	TYR	C	704	SER
A	734	THR	B	708	SER	C	705	VAL
A	735	SER	B	709	ASN	C	706	ALA
A	736	VAL	B	710	ASN	C	707	TYR
A	737	ASP	B	711	SER	C	708	SER
A	738	CYS	B	712	ILE	C	709	ASN
A	739	THR	B	713	ALA	C	710	ASN
A	740	MET	B	715	PRO	C	711	SER
A	741	TYR	B	716	THR	C	712	ILE
A	742	ILE	B	717	ASN	C	713	ALA
A	743	CYS	B	719	THR	C	716	THR
A	745	ASP	B	720	ILE	C	717	ASN
A	746	SER	B	721	SER	C	719	THR
A	747	THR	B	722	VAL	C	720	ILE
A	748	GLU	B	723	THR	C	721	SER
A	750	SER	B	724	THR	C	722	VAL
A	751	ASN	B	725	GLU	C	723	THR
A	752	LEU	B	726	ILE	C	724	THR
A	753	LEU	B	727	LEU	C	725	GLU
A	754	LEU	B	728	PRO	C	726	ILE
A	755	GLN	B	729	VAL	C	727	LEU
A	756	TYR	B	730	SER	C	728	PRO
A	757	GLY	B	731	MET	C	730	SER
A	758	SER	B	732	THR	C	731	MET
A	760	CYS	B	733	LYS	C	732	THR
A	761	THR	B	735	SER	C	733	LYS
A	762	GLN	B	737	ASP	C	735	SER
A	763	LEU	B	738	CYS	C	736	VAL
A	764	ASN	B	739	THR	C	737	ASP
A	765	ARG	B	740	MET	C	738	CYS
A	766	ALA	B	742	ILE	C	739	THR
A	768	THR	B	743	CYS	C	740	MET
A	769	GLY	B	744	GLY	C	742	ILE
A	770	ILE	B	745	ASP	C	743	CYS
A	771	ALA	B	746	SER	C	744	GLY

A	750	SER	B	722	VAL	C	758	SER
A	751	ASN	B	723	THR	C	760	CYS
A	754	LEU	B	724	THR	C	761	THR
A	755	GLN	B	725	GLU	C	764	ASN
A	757	GLY	B	726	ILE	C	765	ARG
A	758	SER	B	727	LEU	C	766	ALA
A	760	CYS	B	728	PRO	C	768	THR
A	761	THR	B	730	SER	C	769	GLY
A	762	GLN	B	731	MET	C	770	ILE
A	764	ASN	B	732	THR	C	772	VAL
A	765	ARG	B	733	LYS	C	773	GLU
A	766	ALA	B	735	SER	C	775	ASP
A	768	THR	B	739	THR	C	776	LYS
A	769	GLY	B	740	MET	C	777	ASN
A	772	VAL	B	744	GLY	C	778	THR
A	773	GLU	B	745	ASP	C	779	GLN
A	775	ASP	B	746	SER	C	780	GLU
A	776	LYS	B	747	THR	C	783	ALA
A	778	THR	B	748	GLU	C	784	GLN
A	779	GLN	B	750	SER	C	785	VAL
A	780	GLU	B	751	ASN	C	786	LYS
A	783	ALA	B	752	LEU	C	787	GLN
A	784	GLN	B	753	LEU	C	790	LYS
A	785	VAL	B	754	LEU	C	791	THR
A	786	LYS	B	755	GLN	C	793	PRO
A	787	GLN	B	756	TYR	C	794	ILE
A	788	ILE	B	757	GLY	C	795	LYS
A	790	LYS	B	758	SER	C	796	ASP
A	791	THR	B	760	CYS	C	798	GLY
A	793	PRO	B	761	THR	C	799	GLY
A	794	ILE	B	762	GLN	C	800	PHE
A	795	LYS	B	764	ASN	C	801	ASN
A	796	ASP	B	765	ARG	C	803	SER
A	798	GLY	B	766	ALA	C	804	GLN
A	799	GLY	B	768	THR	C	807	PRO
A	800	PHE	B	769	GLY	C	808	ASP
A	801	ASN	B	772	VAL	C	809	PRO
A	803	SER	B	773	GLU	C	810	SER
A	804	GLN	B	775	ASP	C	811	LYS
A	807	PRO	B	776	LYS	C	815	ARG
A	808	ASP	B	778	THR	C	817	PHE
A	809	PRO	B	779	GLN	C	820	ASP
A	810	SER	B	780	GLU	C	821	LEU
A	811	LYS	B	782	PHE	C	823	PHE
A	815	ARG	B	783	ALA	C	824	ASN
A	817	PHE	B	784	GLN	C	825	LYS
A	820	ASP	B	785	VAL	C	826	VAL
A	821	LEU	B	786	LYS	C	827	THR
A	823	PHE	B	787	GLN	C	828	LEU
A	824	ASN	B	788	ILE	C	853	GLN
A	825	LYS	B	789	TYR	C	854	LYS
A	826	VAL	B	790	LYS	C	855	PHE
A	827	THR	B	791	THR	C	856	ASN
A	828	LEU	B	793	PRO	C	859	THR
A	853	GLN	B	794	ILE	C	860	VAL
A	854	LYS	B	795	LYS	C	861	LEU
A	855	PHE	B	796	ASP	C	862	PRO
A	856	ASN	B	798	GLY	C	863	PRO
A	860	VAL	B	799	GLY	C	864	LEU
A	861	LEU	B	800	PHE	C	866	THR
A	862	PRO	B	801	ASN	C	867	ASP
A	863	PRO	B	802	PHE	C	868	GLU
A	864	LEU	B	803	SER	C	869	MET
A	866	THR	B	804	GLN	C	870	ILE
A	867	ASP	B	807	PRO	C	872	GLN
A	868	GLU	B	808	ASP	C	873	TYR
A	870	ILE	B	809	PRO	C	875	SER
A	872	GLN	B	810	SER	C	886	TRP
A	873	TYR	B	811	LYS	C	887	THR
A	875	SER	B	815	ARG	C	889	GLY
A	886	TRP	B	817	PHE	C	891	GLY
A	887	THR	B	820	ASP	C	892	ALA
A	888	PHE	B	821	LEU	C	893	ALA
A	890	ALA	B	823	PHE	C	895	GLN
A	891	GLY	B	824	ASN	C	899	ALA
A	892	ALA	B	825	LYS	C	904	TYR
A	893	ALA	B	826	VAL	C	907	ASN
A	895	GLN	B	827	THR	C	908	GLY
A	896	ILE	B	828	LEU	C	909	ILE
A	897	PRO	B	853	GLN	C	910	GLY
A	899	ALA	B	854	LYS	C	912	THR
A	904	TYR	B	855	PHE	C	913	GLN
A	907	ASN	B	856	ASN	C	918	GLU
A	908	GLY	B	859	THR	C	919	ASN
A	909	ILE	B	860	VAL	C	921	LYS
A	910	GLY	B	861	LEU	C	922	LEU
A	912	THR	B	862	PRO	C	925	ASN
A	918	GLU	B	863	PRO	C	926	GLN
A	919	ASN	B	864	LEU	C	928	ASN
A	920	GLN	B	866	THR	C	929	SER

A	788	ILE	B	754	LEU	C	785	VAL
A	790	LYS	B	755	GLN	C	786	LYS
A	791	THR	B	756	TYR	C	787	GLN
A	792	PRO	B	757	GLY	C	790	LYS
A	793	PRO	B	758	SER	C	791	THR
A	794	ILE	B	760	CYS	C	792	PRO
A	795	LYS	B	761	THR	C	793	PRO
A	796	ASP	B	764	ASN	C	794	ILE
A	798	GLY	B	765	ARG	C	795	LYS
A	799	GLY	B	768	THR	C	796	ASP
A	801	ASN	B	769	GLY	C	798	GLY
A	803	SER	B	772	VAL	C	799	GLY
A	804	GLN	B	773	GLU	C	800	PHE
A	807	PRO	B	775	ASP	C	801	ASN
A	808	ASP	B	776	LYS	C	803	SER
A	809	PRO	B	779	GLN	C	804	GLN
A	810	SER	B	780	GLU	C	807	PRO
A	811	LYS	B	783	ALA	C	808	ASP
A	815	ARG	B	784	GLN	C	809	PRO
A	817	PHE	B	786	LYS	C	810	SER
A	820	ASP	B	787	GLN	C	811	LYS
A	821	LEU	B	788	ILE	C	815	ARG
A	823	PHE	B	790	LYS	C	817	PHE
A	824	ASN	B	791	THR	C	820	ASP
A	825	LYS	B	793	PRO	C	821	LEU
A	826	VAL	B	794	ILE	C	823	PHE
A	827	THR	B	795	LYS	C	824	ASN
A	828	LEU	B	796	ASP	C	825	LYS
A	853	GLN	B	798	GLY	C	826	VAL
A	854	LYS	B	799	GLY	C	827	THR
A	855	PHE	B	801	ASN	C	828	LEU
A	856	ASN	B	803	SER	C	853	GLN
A	860	VAL	B	804	GLN	C	854	LYS
A	861	LEU	B	807	PRO	C	855	PHE
A	862	PRO	B	808	ASP	C	856	ASN
A	863	PRO	B	809	PRO	C	859	THR
A	864	LEU	B	810	SER	C	860	VAL
A	866	THR	B	811	LYS	C	861	LEU
A	867	ASP	B	815	ARG	C	862	PRO
A	868	GLU	B	817	PHE	C	863	PRO
A	870	ILE	B	820	ASP	C	864	LEU
A	872	GLN	B	821	LEU	C	866	THR
A	873	TYR	B	823	PHE	C	867	ASP
A	875	SER	B	824	ASN	C	868	GLU
A	886	TRP	B	825	LYS	C	869	MET
A	887	THR	B	827	THR	C	870	ILE
A	888	PHE	B	828	LEU	C	872	GLN
A	891	GLY	B	853	GLN	C	873	TYR
A	892	ALA	B	854	LYS	C	875	SER
A	893	ALA	B	855	PHE	C	886	TRP
A	895	GLN	B	856	ASN	C	887	THR
A	896	ILE	B	860	VAL	C	889	GLY
A	899	ALA	B	861	LEU	C	891	GLY
A	904	TYR	B	862	PRO	C	892	ALA
A	907	ASN	B	863	PRO	C	893	ALA
A	908	GLY	B	864	LEU	C	895	GLN
A	909	ILE	B	866	THR	C	899	ALA
A	910	GLY	B	867	ASP	C	904	TYR
A	912	THR	B	868	GLU	C	907	ASN
A	918	GLU	B	869	MET	C	908	GLY
A	919	ASN	B	870	ILE	C	909	ILE
A	921	LYS	B	872	GLN	C	910	GLY
A	922	LEU	B	873	TYR	C	912	THR
A	925	ASN	B	875	SER	C	913	GLN
A	926	GLN	B	886	TRP	C	918	GLU
A	928	ASN	B	887	THR	C	919	ASN
A	929	SER	B	888	PHE	C	921	LYS
A	932	GLY	B	891	GLY	C	922	LEU
A	933	LYS	B	892	ALA	C	925	ASN
A	934	ILE	B	893	ALA	C	926	GLN
A	935	GLN	B	895	GLN	C	928	ASN
A	936	ASP	B	897	PRO	C	929	SER
A	937	SER	B	899	ALA	C	930	ALA
A	939	SER	B	904	TYR	C	932	GLY
A	940	SER	B	907	ASN	C	933	LYS
A	941	THR	B	908	GLY	C	934	ILE
A	942	ALA	B	909	ILE	C	935	GLN
A	943	SER	B	910	GLY	C	936	ASP
A	944	ALA	B	912	THR	C	937	SER
A	946	GLY	B	918	GLU	C	938	LEU
A	947	LYS	B	919	ASN	C	939	SER
A	949	GLN	B	921	LYS	C	940	SER
A	950	ASP	B	922	LEU	C	941	THR
A	951	VAL	B	925	ASN	C	942	ALA
A	952	VAL	B	926	GLN	C	943	SER
A	953	ASN	B	928	ASN	C	944	ALA
A	954	GLN	B	929	SER	C	946	GLY
A	956	ALA	B	930	ALA	C	947	LYS
A	957	GLN	B	932	GLY	C	949	GLN
A	958	ALA	B	933	LYS	C	950	ASP

A	772	VAL	B	747	THR	C	745	ASP
A	773	GLU	B	748	GLU	C	746	SER
A	774	GLN	B	750	SER	C	747	THR
A	775	ASP	B	751	ASN	C	748	GLU
A	776	LYS	B	752	LEU	C	750	SER
A	778	THR	B	753	LEU	C	751	ASN
A	779	GLN	B	754	LEU	C	752	LEU
A	780	GLU	B	755	GLN	C	753	LEU
A	782	PHE	B	756	TYR	C	754	LEU
A	783	ALA	B	757	GLY	C	755	GLN
A	784	GLN	B	758	SER	C	756	TYR
A	785	VAL	B	759	PHE	C	757	GLY
A	786	LYS	B	760	CYS	C	758	SER
A	787	GLN	B	761	THR	C	759	PHE
A	788	ILE	B	762	GLN	C	760	CYS
A	789	TYR	B	763	LEU	C	761	THR
A	790	LYS	B	764	ASN	C	762	GLN
A	791	THR	B	765	ARG	C	763	LEU
A	792	PRO	B	766	ALA	C	764	ASN
A	793	PRO	B	767	LEU	C	765	ARG
A	794	ILE	B	768	THR	C	766	ALA
A	795	LYS	B	769	GLY	C	767	LEU
A	796	ASP	B	770	ILE	C	768	THR
A	797	PHE	B	771	ALA	C	769	GLY
A	798	GLY	B	772	VAL	C	770	ILE
A	799	GLY	B	773	GLU	C	771	ALA
A	800	PHE	B	774	GLN	C	772	VAL
A	801	ASN	B	775	ASP	C	773	GLU
A	802	PHE	B	776	LYS	C	775	ASP
A	803	SER	B	777	ASN	C	776	LYS
A	804	GLN	B	778	THR	C	777	ASN
A	807	PRO	B	779	GLN	C	778	THR
A	808	ASP	B	780	GLU	C	779	GLN
A	809	PRO	B	782	PHE	C	780	GLU
A	810	SER	B	783	ALA	C	782	PHE
A	811	LYS	B	784	GLN	C	783	ALA
A	815	ARG	B	785	VAL	C	784	GLN
A	817	PHE	B	786	LYS	C	785	VAL
A	819	GLU	B	787	GLN	C	786	LYS
A	820	ASP	B	788	ILE	C	787	GLN
A	821	LEU	B	789	TYR	C	788	ILE
A	823	PHE	B	790	LYS	C	789	TYR
A	824	ASN	B	791	THR	C	790	LYS
A	825	LYS	B	792	PRO	C	791	THR
A	826	VAL	B	793	PRO	C	792	PRO
A	827	THR	B	794	ILE	C	793	PRO
A	828	LEU	B	795	LYS	C	794	ILE
A	853	GLN	B	796	ASP	C	795	LYS
A	854	LYS	B	797	PHE	C	796	ASP
A	855	PHE	B	798	GLY	C	797	PHE
A	856	ASN	B	799	GLY	C	798	GLY
A	857	GLY	B	800	PHE	C	799	GLY
A	858	LEU	B	801	ASN	C	800	PHE
A	859	THR	B	802	PHE	C	801	ASN
A	860	VAL	B	803	SER	C	802	PHE
A	861	LEU	B	804	GLN	C	803	SER
A	862	PRO	B	807	PRO	C	804	GLN
A	863	PRO	B	808	ASP	C	805	ILE
A	864	LEU	B	809	PRO	C	807	PRO
A	865	LEU	B	810	SER	C	808	ASP
A	866	THR	B	811	LYS	C	809	PRO
A	867	ASP	B	815	ARG	C	810	SER
A	868	GLU	B	817	PHE	C	811	LYS
A	869	MET	B	819	GLU	C	815	ARG
A	870	ILE	B	820	ASP	C	817	PHE
A	871	ALA	B	821	LEU	C	818	ILE
A	872	GLN	B	823	PHE	C	819	GLU
A	873	TYR	B	824	ASN	C	820	ASP
A	875	SER	B	825	LYS	C	821	LEU
A	886	TRP	B	826	VAL	C	823	PHE
A	887	THR	B	827	THR	C	824	ASN
A	888	PHE	B	828	LEU	C	825	LYS
A	889	GLY	B	853	GLN	C	826	VAL
A	890	ALA	B	854	LYS	C	827	THR
A	891	GLY	B	855	PHE	C	828	LEU
A	892	ALA	B	856	ASN	C	853	GLN
A	893	ALA	B	857	GLY	C	854	LYS
A	894	LEU	B	859	THR	C	855	PHE
A	895	GLN	B	860	VAL	C	856	ASN
A	896	ILE	B	861	LEU	C	857	GLY
A	897	PRO	B	862	PRO	C	858	LEU
A	899	ALA	B	863	PRO	C	859	THR
A	900	MET	B	864	LEU	C	860	VAL
A	904	TYR	B	865	LEU	C	861	LEU
A	907	ASN	B	866	THR	C	862	PRO
A	908	GLY	B	867	ASP	C	863	PRO
A	909	ILE	B	868	GLU	C	864	LEU
A	910	GLY	B	869	MET	C	865	LEU
A	912	THR	B	870	ILE	C	866	THR
A	913	GLN	B	871	ALA	C	867	ASP

A	921	LYS	B	867	ASP	C	930	ALA
A	922	LEU	B	868	GLU	C	932	GLY
A	925	ASN	B	869	MET	C	933	LYS
A	926	GLN	B	870	ILE	C	934	ILE
A	928	ASN	B	872	GLN	C	935	GLN
A	929	SER	B	873	TYR	C	936	ASP
A	932	GLY	B	875	SER	C	937	SER
A	933	LYS	B	886	TRP	C	938	LEU
A	934	ILE	B	887	THR	C	939	SER
A	935	GLN	B	888	PHE	C	940	SER
A	936	ASP	B	891	GLY	C	941	THR
A	937	SER	B	892	ALA	C	942	ALA
A	939	SER	B	893	ALA	C	943	SER
A	940	SER	B	894	LEU	C	944	ALA
A	941	THR	B	895	GLN	C	946	GLY
A	942	ALA	B	897	PRO	C	947	LYS
A	943	SER	B	899	ALA	C	949	GLN
A	944	ALA	B	904	TYR	C	950	ASP
A	946	GLY	B	907	ASN	C	951	VAL
A	947	LYS	B	908	GLY	C	952	VAL
A	949	GLN	B	909	ILE	C	953	ASN
A	950	ASP	B	910	GLY	C	954	GLN
A	951	VAL	B	912	THR	C	955	ASN
A	952	VAL	B	918	GLU	C	956	ALA
A	953	ASN	B	919	ASN	C	957	GLN
A	954	GLN	B	921	LYS	C	958	ALA
A	956	ALA	B	922	LEU	C	959	LEU
A	957	GLN	B	925	ASN	C	960	ASN
A	958	ALA	B	926	GLN	C	964	LYS
A	959	LEU	B	928	ASN	C	967	SER
A	960	ASN	B	929	SER	C	969	ASN
A	964	LYS	B	930	ALA	C	971	GLY
A	966	LEU	B	932	GLY	C	972	ALA
A	967	SER	B	933	LYS	C	973	ILE
A	968	SER	B	934	ILE	C	974	SER
A	969	ASN	B	935	GLN	C	976	VAL
A	970	PHE	B	936	ASP	C	977	LEU
A	971	GLY	B	937	SER	C	978	ASN
A	972	ALA	B	938	LEU	C	979	ASP
A	973	ILE	B	939	SER	C	981	LEU
A	974	SER	B	940	SER	C	982	SER
A	975	SER	B	941	THR	C	983	ARG
A	977	LEU	B	942	ALA	C	984	LEU
A	978	ASN	B	943	SER	C	985	ASP
A	979	ASP	B	944	ALA	C	986	PRO
A	981	LEU	B	946	GLY	C	987	PRO
A	982	SER	B	947	LYS	C	988	GLU
A	983	ARG	B	949	GLN	C	990	GLU
A	985	ASP	B	950	ASP	C	991	VAL
A	986	PRO	B	951	VAL	C	992	GLN
A	987	PRO	B	952	VAL	C	995	ARG
A	988	GLU	B	953	ASN	C	998	THR
A	990	GLU	B	954	GLN	C	1000	ARG
A	991	VAL	B	955	ASN	C	1002	GLN
A	992	GLN	B	956	ALA	C	1005	GLN
A	995	ARG	B	957	GLN	C	1006	THR
A	998	THR	B	958	ALA	C	1009	THR
A	1000	ARG	B	959	LEU	C	1010	GLN
A	1002	GLN	B	960	ASN	C	1012	LEU
A	1005	GLN	B	961	THR	C	1013	ILE
A	1006	THR	B	963	VAL	C	1014	ARG
A	1009	THR	B	964	LYS	C	1016	ALA
A	1010	GLN	B	966	LEU	C	1017	GLU
A	1012	LEU	B	967	SER	C	1019	ARG
A	1013	ILE	B	969	ASN	C	1020	ALA
A	1014	ARG	B	971	GLY	C	1021	SER
A	1016	ALA	B	972	ALA	C	1023	ASN
A	1017	GLU	B	973	ILE	C	1024	LEU
A	1018	ILE	B	974	SER	C	1026	ALA
A	1019	ARG	B	975	SER	C	1027	THR
A	1020	ALA	B	976	VAL	C	1028	LYS
A	1021	SER	B	977	LEU	C	1030	SER
A	1023	ASN	B	978	ASN	C	1036	GLN
A	1024	LEU	B	979	ASP	C	1037	SER
A	1026	ALA	B	981	LEU	C	1038	LYS
A	1027	THR	B	983	ARG	C	1039	ARG
A	1028	LYS	B	984	LEU	C	1040	VAL
A	1030	SER	B	985	ASP	C	1041	ASP
A	1031	GLU	B	987	PRO	C	1042	PHE
A	1036	GLN	B	988	GLU	C	1044	GLY
A	1037	SER	B	990	GLU	C	1045	LYS
A	1038	LYS	B	991	VAL	C	1046	GLY
A	1039	ARG	B	992	GLN	C	1047	TYR
A	1040	VAL	B	994	ASP	C	1048	HIS
A	1041	ASP	B	995	ARG	C	1057	PRO
A	1042	PHE	B	998	THR	C	1058	HIS
A	1044	GLY	B	1000	ARG	C	1068	VAL
A	1045	LYS	B	1005	GLN	C	1069	PRO
A	1046	GLY	B	1006	THR	C	1070	ALA
A	1047	TYR	B	1007	TYR	C	1071	GLN

A	959	LEU	B	934	ILE	C	951	VAL
A	960	ASN	B	935	GLN	C	952	VAL
A	964	LYS	B	936	ASP	C	953	ASN
A	966	LEU	B	937	SER	C	954	GLN
A	967	SER	B	939	SER	C	955	ASN
A	968	SER	B	940	SER	C	956	ALA
A	969	ASN	B	941	THR	C	957	GLN
A	970	PHE	B	942	ALA	C	959	LEU
A	971	GLY	B	943	SER	C	960	ASN
A	972	ALA	B	944	ALA	C	964	LYS
A	973	ILE	B	946	GLY	C	967	SER
A	974	SER	B	947	LYS	C	969	ASN
A	975	SER	B	949	GLN	C	971	GLY
A	978	ASN	B	950	ASP	C	972	ALA
A	979	ASP	B	952	VAL	C	973	ILE
A	981	LEU	B	953	ASN	C	974	SER
A	982	SER	B	954	GLN	C	976	VAL
A	983	ARG	B	955	ASN	C	977	LEU
A	985	ASP	B	956	ALA	C	978	ASN
A	986	PRO	B	957	GLN	C	979	ASP
A	987	PRO	B	959	LEU	C	981	LEU
A	988	GLU	B	960	ASN	C	982	SER
A	990	GLU	B	964	LYS	C	983	ARG
A	991	VAL	B	967	SER	C	984	LEU
A	992	GLN	B	969	ASN	C	985	ASP
A	995	ARG	B	972	ALA	C	986	PRO
A	998	THR	B	973	ILE	C	987	PRO
A	1000	ARG	B	974	SER	C	988	GLU
A	1002	GLN	B	975	SER	C	990	GLU
A	1005	GLN	B	976	VAL	C	991	VAL
A	1006	THR	B	977	LEU	C	992	GLN
A	1009	THR	B	978	ASN	C	994	ASP
A	1010	GLN	B	979	ASP	C	995	ARG
A	1012	LEU	B	981	LEU	C	998	THR
A	1013	ILE	B	982	SER	C	1000	ARG
A	1014	ARG	B	983	ARG	C	1005	GLN
A	1016	ALA	B	984	LEU	C	1006	THR
A	1017	GLU	B	985	ASP	C	1009	THR
A	1018	ILE	B	987	PRO	C	1010	GLN
A	1019	ARG	B	988	GLU	C	1012	LEU
A	1020	ALA	B	990	GLU	C	1013	ILE
A	1021	SER	B	991	VAL	C	1014	ARG
A	1023	ASN	B	992	GLN	C	1016	ALA
A	1024	LEU	B	994	ASP	C	1017	GLU
A	1026	ALA	B	995	ARG	C	1019	ARG
A	1027	THR	B	998	THR	C	1020	ALA
A	1028	LYS	B	1000	ARG	C	1021	SER
A	1030	SER	B	1005	GLN	C	1023	ASN
A	1036	GLN	B	1009	THR	C	1024	LEU
A	1037	SER	B	1010	GLN	C	1026	ALA
A	1038	LYS	B	1012	LEU	C	1027	THR
A	1039	ARG	B	1013	ILE	C	1028	LYS
A	1040	VAL	B	1014	ARG	C	1030	SER
A	1041	ASP	B	1016	ALA	C	1036	GLN
A	1042	PHE	B	1017	GLU	C	1037	SER
A	1044	GLY	B	1019	ARG	C	1038	LYS
A	1045	LYS	B	1020	ALA	C	1039	ARG
A	1046	GLY	B	1021	SER	C	1040	VAL
A	1047	TYR	B	1023	ASN	C	1041	ASP
A	1048	HIS	B	1024	LEU	C	1042	PHE
A	1057	PRO	B	1026	ALA	C	1044	GLY
A	1058	HIS	B	1027	THR	C	1045	LYS
A	1068	VAL	B	1028	LYS	C	1046	GLY
A	1069	PRO	B	1030	SER	C	1047	TYR
A	1070	ALA	B	1036	GLN	C	1048	HIS
A	1071	GLN	B	1037	SER	C	1057	PRO
A	1072	GLU	B	1038	LYS	C	1058	HIS
A	1073	LYS	B	1039	ARG	C	1068	VAL
A	1074	ASN	B	1040	VAL	C	1069	PRO
A	1076	THR	B	1041	ASP	C	1070	ALA
A	1083	HIS	B	1042	PHE	C	1071	GLN
A	1084	ASP	B	1044	GLY	C	1072	GLU
A	1085	GLY	B	1045	LYS	C	1073	LYS
A	1086	LYS	B	1046	GLY	C	1074	ASN
A	1091	ARG	B	1047	TYR	C	1076	THR
A	1092	GLU	B	1056	ALA	C	1079	PRO
A	1098	ASN	B	1057	PRO	C	1082	CYS
A	1099	GLY	B	1058	HIS	C	1083	HIS
A	1100	THR	B	1068	VAL	C	1084	ASP
A	1101	HIS	B	1069	PRO	C	1085	GLY
A	1102	TRP	B	1070	ALA	C	1086	LYS
A	1103	PHE	B	1071	GLN	C	1091	ARG
A	1107	ARG	B	1072	GLU	C	1092	GLU
A	1108	ASN	B	1073	LYS	C	1093	GLY
A	1109	PHE	B	1074	ASN	C	1098	ASN
A	1110	TYR	B	1075	PHE	C	1099	GLY
A	1111	GLU	B	1076	THR	C	1100	THR
A	1112	PRO	B	1082	CYS	C	1101	HIS
A	1113	GLN	B	1083	HIS	C	1102	TRP
A	1114	ILE	B	1084	ASP	C	1103	PHE

A	917	TYR	B	872	GLN	C	868	GLU
A	918	GLU	B	873	TYR	C	869	MET
A	919	ASN	B	875	SER	C	870	ILE
A	920	GLN	B	879	ALA	C	871	ALA
A	921	LYS	B	884	SER	C	872	GLN
A	922	LEU	B	886	TRP	C	873	TYR
A	924	ALA	B	887	THR	C	875	SER
A	925	ASN	B	888	PHE	C	884	SER
A	926	GLN	B	889	GLY	C	886	TRP
A	928	ASN	B	891	GLY	C	887	THR
A	929	SER	B	892	ALA	C	889	GLY
A	930	ALA	B	893	ALA	C	890	ALA
A	931	ILE	B	894	LEU	C	891	GLY
A	932	GLY	B	895	GLN	C	892	ALA
A	933	LYS	B	896	ILE	C	893	ALA
A	934	ILE	B	897	PRO	C	894	LEU
A	935	GLN	B	899	ALA	C	895	GLN
A	936	ASP	B	900	MET	C	896	ILE
A	937	SER	B	902	MET	C	897	PRO
A	938	LEU	B	904	TYR	C	899	ALA
A	939	SER	B	907	ASN	C	900	MET
A	940	SER	B	908	GLY	C	904	TYR
A	941	THR	B	909	ILE	C	907	ASN
A	942	ALA	B	910	GLY	C	908	GLY
A	943	SER	B	912	THR	C	909	ILE
A	944	ALA	B	913	GLN	C	910	GLY
A	946	GLY	B	917	TYR	C	912	THR
A	947	LYS	B	918	GLU	C	913	GLN
A	949	GLN	B	919	ASN	C	915	VAL
A	950	ASP	B	920	GLN	C	917	TYR
A	951	VAL	B	921	LYS	C	918	GLU
A	952	VAL	B	922	LEU	C	919	ASN
A	953	ASN	B	924	ALA	C	920	GLN
A	954	GLN	B	925	ASN	C	921	LYS
A	955	ASN	B	926	GLN	C	922	LEU
A	956	ALA	B	928	ASN	C	924	ALA
A	957	GLN	B	929	SER	C	925	ASN
A	958	ALA	B	930	ALA	C	926	GLN
A	959	LEU	B	932	GLY	C	928	ASN
A	960	ASN	B	933	LYS	C	929	SER
A	961	THR	B	934	ILE	C	930	ALA
A	962	LEU	B	935	GLN	C	931	ILE
A	963	VAL	B	936	ASP	C	932	GLY
A	964	LYS	B	937	SER	C	933	LYS
A	965	GLN	B	938	LEU	C	934	ILE
A	966	LEU	B	939	SER	C	935	GLN
A	967	SER	B	940	SER	C	936	ASP
A	968	SER	B	941	THR	C	937	SER
A	969	ASN	B	942	ALA	C	938	LEU
A	970	PHE	B	943	SER	C	939	SER
A	971	GLY	B	944	ALA	C	940	SER
A	972	ALA	B	945	LEU	C	941	THR
A	973	ILE	B	946	GLY	C	942	ALA
A	974	SER	B	947	LYS	C	943	SER
A	975	SER	B	949	GLN	C	944	ALA
A	976	VAL	B	950	ASP	C	945	LEU
A	977	LEU	B	951	VAL	C	946	GLY
A	978	ASN	B	952	VAL	C	947	LYS
A	979	ASP	B	953	ASN	C	949	GLN
A	981	LEU	B	954	GLN	C	950	ASP
A	982	SER	B	955	ASN	C	951	VAL
A	983	ARG	B	956	ALA	C	952	VAL
A	984	LEU	B	957	GLN	C	953	ASN
A	985	ASP	B	958	ALA	C	954	GLN
A	986	PRO	B	959	LEU	C	955	ASN
A	987	PRO	B	960	ASN	C	956	ALA
A	988	GLU	B	961	THR	C	957	GLN
A	990	GLU	B	963	VAL	C	958	ALA
A	991	VAL	B	964	LYS	C	959	LEU
A	992	GLN	B	965	GLN	C	960	ASN
A	994	ASP	B	966	LEU	C	961	THR
A	995	ARG	B	967	SER	C	962	LEU
A	996	LEU	B	968	SER	C	963	VAL
A	997	ILE	B	969	ASN	C	964	LYS
A	998	THR	B	970	PHE	C	966	LEU
A	999	GLY	B	971	GLY	C	967	SER
A	1000	ARG	B	972	ALA	C	968	SER
A	1001	LEU	B	973	ILE	C	969	ASN
A	1002	GLN	B	974	SER	C	970	PHE
A	1003	SER	B	975	SER	C	971	GLY
A	1004	LEU	B	976	VAL	C	972	ALA
A	1005	GLN	B	977	LEU	C	973	ILE
A	1006	THR	B	978	ASN	C	974	SER
A	1007	TYR	B	979	ASP	C	975	SER
A	1009	THR	B	981	LEU	C	976	VAL
A	1010	GLN	B	982	SER	C	977	LEU
A	1012	LEU	B	983	ARG	C	978	ASN
A	1013	ILE	B	984	LEU	C	979	ASP
A	1014	ARG	B	985	ASP	C	981	LEU
A	1016	ALA	B	986	PRO	C	982	SER

A	1048	HIS	B	1009	THR	C	1072	GLU
A	1057	PRO	B	1010	GLN	C	1073	LYS
A	1058	HIS	B	1012	LEU	C	1074	ASN
A	1068	VAL	B	1013	ILE	C	1075	PHE
A	1069	PRO	B	1014	ARG	C	1076	THR
A	1070	ALA	B	1016	ALA	C	1079	PRO
A	1071	GLN	B	1017	GLU	C	1082	CYS
A	1072	GLU	B	1018	ILE	C	1083	HIS
A	1073	LYS	B	1019	ARG	C	1084	ASP
A	1074	ASN	B	1020	ALA	C	1085	GLY
A	1076	THR	B	1021	SER	C	1086	LYS
A	1083	HIS	B	1023	ASN	C	1091	ARG
A	1084	ASP	B	1024	LEU	C	1092	GLU
A	1085	GLY	B	1026	ALA	C	1093	GLY
A	1086	LYS	B	1027	THR	C	1098	ASN
A	1091	ARG	B	1028	LYS	C	1099	GLY
A	1092	GLU	B	1030	SER	C	1100	THR
A	1093	GLY	B	1036	GLN	C	1101	HIS
A	1098	ASN	B	1037	SER	C	1102	TRP
A	1099	GLY	B	1038	LYS	C	1103	PHE
A	1100	THR	B	1039	ARG	C	1104	VAL
A	1101	HIS	B	1040	VAL	C	1106	GLN
A	1102	TRP	B	1041	ASP	C	1107	ARG
A	1103	PHE	B	1042	PHE	C	1108	ASN
A	1107	ARG	B	1044	GLY	C	1109	PHE
A	1108	ASN	B	1045	LYS	C	1110	TYR
A	1109	PHE	B	1046	GLY	C	1111	GLU
A	1110	TYR	B	1047	TYR	C	1112	PRO
A	1111	GLU	B	1056	ALA	C	1113	GLN
A	1112	PRO	B	1057	PRO	C	1114	ILE
A	1113	GLN	B	1058	HIS	C	1116	THR
A	1114	ILE	B	1068	VAL	C	1117	THR
A	1116	THR	B	1069	PRO	C	1118	ASP
A	1117	THR	B	1070	ALA	C	1119	ASN
A	1118	ASP	B	1071	GLN	C	1121	PHE
A	1119	ASN	B	1072	GLU	C	1122	VAL
A	1121	PHE	B	1073	LYS	C	1123	SER
A	1122	VAL	B	1074	ASN	C	1124	GLY
A	1123	SER	B	1075	PHE	C	1125	ASN
A	1124	GLY	B	1076	THR	C	1126	CYS
A	1125	ASN	B	1082	CYS	C	1127	ASP
A	1126	CYS	B	1083	HIS	C	1128	VAL
A	1127	ASP	B	1084	ASP	C	1129	VAL
A	1128	VAL	B	1085	GLY	C	1130	ILE
A	1129	VAL	B	1086	LYS	C	1131	GLY
A	1130	ILE	B	1088	HIS	C	1132	ILE
A	1131	GLY	B	1091	ARG	C	1133	VAL
A	1132	ILE	B	1092	GLU	C	1134	ASN
A	1133	VAL	B	1097	SER	C	1135	ASN
A	1134	ASN	B	1098	ASN	C	1136	THR
A	1135	ASN	B	1099	GLY	C	1138	TYR
A	1136	THR	B	1100	THR	C	1139	ASP
A	1137	VAL	B	1101	HIS	C	1140	PRO
A	1138	TYR	B	1102	TRP	C	1141	LEU
A	1139	ASP	B	1103	PHE	C	1142	GLN
A	1140	PRO	B	1106	GLN	C	1143	PRO
A	1141	LEU	B	1107	ARG	C	1144	GLU
A	1142	GLN	B	1108	ASN	C	1145	LEU
A	1143	PRO	B	1109	PHE	C	1146	ASP
A	1144	GLU	B	1110	TYR			
A	1145	LEU	B	1111	GLU			
A	1146	ASP	B	1112	PRO			
			B	1113	GLN			
			B	1114	ILE			
			B	1116	THR			
			B	1117	THR			
			B	1118	ASP			
			B	1119	ASN			
			B	1120	THR			
			B	1121	PHE			
			B	1122	VAL			
			B	1124	GLY			
			B	1125	ASN			
			B	1126	CYS			
			B	1127	ASP			
			B	1128	VAL			
			B	1129	VAL			
			B	1130	ILE			
			B	1131	GLY			
			B	1132	ILE			
			B	1133	VAL			
			B	1134	ASN			
			B	1135	ASN			
			B	1136	THR			
			B	1138	TYR			
			B	1139	ASP			
			B	1140	PRO			
			B	1141	LEU			
			B	1142	GLN			
			B	1143	PRO			

A	1116	THR	B	1085	GLY	C	1106	GLN
A	1117	THR	B	1086	LYS	C	1107	ARG
A	1118	ASP	B	1088	HIS	C	1108	ASN
A	1119	ASN	B	1091	ARG	C	1109	PHE
A	1121	PHE	B	1092	GLU	C	1110	TYR
A	1122	VAL	B	1097	SER	C	1111	GLU
A	1123	SER	B	1098	ASN	C	1112	PRO
A	1124	GLY	B	1099	GLY	C	1113	GLN
A	1125	ASN	B	1100	THR	C	1114	ILE
A	1126	CYS	B	1101	HIS	C	1116	THR
A	1127	ASP	B	1102	TRP	C	1117	THR
A	1128	VAL	B	1103	PHE	C	1118	ASP
A	1129	VAL	B	1106	GLN	C	1119	ASN
A	1130	ILE	B	1107	ARG	C	1121	PHE
A	1131	GLY	B	1108	ASN	C	1122	VAL
A	1132	ILE	B	1109	PHE	C	1123	SER
A	1133	VAL	B	1110	TYR	C	1124	GLY
A	1134	ASN	B	1111	GLU	C	1125	ASN
A	1135	ASN	B	1112	PRO	C	1126	CYS
A	1136	THR	B	1113	GLN	C	1127	ASP
A	1137	VAL	B	1114	ILE	C	1128	VAL
A	1138	TYR	B	1116	THR	C	1129	VAL
A	1139	ASP	B	1117	THR	C	1130	ILE
A	1140	PRO	B	1118	ASP	C	1131	GLY
A	1141	LEU	B	1119	ASN	C	1132	ILE
A	1142	GLN	B	1121	PHE	C	1133	VAL
A	1143	PRO	B	1122	VAL	C	1134	ASN
A	1144	GLU	B	1124	GLY	C	1135	ASN
A	1145	LEU	B	1125	ASN	C	1136	THR
A	1146	ASP	B	1126	CYS	C	1138	TYR
			B	1127	ASP	C	1139	ASP
			B	1128	VAL	C	1140	PRO
			B	1129	VAL	C	1141	LEU
			B	1130	ILE	C	1142	GLN
			B	1131	GLY	C	1143	PRO
			B	1132	ILE	C	1144	GLU
			B	1133	VAL	C	1145	LEU
			B	1134	ASN	C	1146	ASP
			B	1135	ASN			
			B	1136	THR			
			B	1138	TYR			
			B	1139	ASP			
			B	1140	PRO			
			B	1141	LEU			
			B	1142	GLN			
			B	1143	PRO			
			B	1144	GLU			
			B	1145	LEU			
			B	1146	ASP			

A	1017	GLU	B	987	PRO	C	983	ARG
A	1018	ILE	B	988	GLU	C	984	LEU
A	1019	ARG	B	990	GLU	C	985	ASP
A	1020	ALA	B	991	VAL	C	986	PRO
A	1021	SER	B	992	GLN	C	987	PRO
A	1023	ASN	B	993	ILE	C	988	GLU
A	1024	LEU	B	994	ASP	C	990	GLU
A	1026	ALA	B	995	ARG	C	991	VAL
A	1027	THR	B	997	ILE	C	992	GLN
A	1028	LYS	B	998	THR	C	993	ILE
A	1030	SER	B	999	GLY	C	994	ASP
A	1031	GLU	B	1000	ARG	C	995	ARG
A	1036	GLN	B	1001	LEU	C	996	LEU
A	1037	SER	B	1002	GLN	C	998	THR
A	1038	LYS	B	1003	SER	C	999	GLY
A	1039	ARG	B	1004	LEU	C	1000	ARG
A	1040	VAL	B	1005	GLN	C	1002	GLN
A	1041	ASP	B	1006	THR	C	1003	SER
A	1042	PHE	B	1007	TYR	C	1005	GLN
A	1044	GLY	B	1008	VAL	C	1006	THR
A	1045	LYS	B	1009	THR	C	1007	TYR
A	1046	GLY	B	1010	GLN	C	1008	VAL
A	1047	TYR	B	1011	GLN	C	1009	THR
A	1048	HIS	B	1012	LEU	C	1010	GLN
A	1057	PRO	B	1013	ILE	C	1012	LEU
A	1058	HIS	B	1014	ARG	C	1013	ILE
A	1061	VAL	B	1015	ALA	C	1014	ARG
A	1068	VAL	B	1016	ALA	C	1016	ALA
A	1069	PRO	B	1017	GLU	C	1017	GLU
A	1070	ALA	B	1018	ILE	C	1019	ARG
A	1071	GLN	B	1019	ARG	C	1020	ALA
A	1072	GLU	B	1020	ALA	C	1021	SER
A	1073	LYS	B	1021	SER	C	1023	ASN
A	1074	ASN	B	1023	ASN	C	1024	LEU
A	1075	PHE	B	1024	LEU	C	1026	ALA
A	1076	THR	B	1026	ALA	C	1027	THR
A	1077	THR	B	1027	THR	C	1028	LYS
A	1079	PRO	B	1028	LYS	C	1030	SER
A	1082	CYS	B	1030	SER	C	1031	GLU
A	1083	HIS	B	1031	GLU	C	1036	GLN
A	1084	ASP	B	1035	GLY	C	1037	SER
A	1085	GLY	B	1036	GLN	C	1038	LYS
A	1086	LYS	B	1037	SER	C	1039	ARG
A	1088	HIS	B	1038	LYS	C	1040	VAL
A	1090	PRO	B	1039	ARG	C	1041	ASP
A	1091	ARG	B	1040	VAL	C	1042	PHE
A	1092	GLU	B	1041	ASP	C	1043	CYS
A	1093	GLY	B	1042	PHE	C	1044	GLY
A	1098	ASN	B	1043	CYS	C	1045	LYS
A	1099	GLY	B	1044	GLY	C	1046	GLY
A	1100	THR	B	1045	LYS	C	1047	TYR
A	1101	HIS	B	1046	GLY	C	1048	HIS
A	1102	TRP	B	1047	TYR	C	1057	PRO
A	1103	PHE	B	1048	HIS	C	1058	HIS
A	1106	GLN	B	1055	SER	C	1061	VAL
A	1107	ARG	B	1056	ALA	C	1068	VAL
A	1108	ASN	B	1057	PRO	C	1069	PRO
A	1109	PHE	B	1058	HIS	C	1070	ALA
A	1110	TYR	B	1061	VAL	C	1071	GLN
A	1111	GLU	B	1068	VAL	C	1072	GLU
A	1112	PRO	B	1069	PRO	C	1073	LYS
A	1113	GLN	B	1070	ALA	C	1074	ASN
A	1114	ILE	B	1071	GLN	C	1075	PHE
A	1115	ILE	B	1072	GLU	C	1076	THR
A	1116	THR	B	1073	LYS	C	1077	THR
A	1117	THR	B	1074	ASN	C	1079	PRO
A	1118	ASP	B	1075	PHE	C	1082	CYS
A	1119	ASN	B	1076	THR	C	1083	HIS
A	1120	THR	B	1077	THR	C	1084	ASP
A	1121	PHE	B	1079	PRO	C	1085	GLY
A	1122	VAL	B	1083	HIS	C	1086	LYS
A	1123	SER	B	1084	ASP	C	1088	HIS
A	1124	GLY	B	1085	GLY	C	1090	PRO
A	1125	ASN	B	1086	LYS	C	1091	ARG
A	1126	CYS	B	1088	HIS	C	1092	GLU
A	1127	ASP	B	1090	PRO	C	1093	GLY
A	1128	VAL	B	1091	ARG	C	1094	VAL
A	1129	VAL	B	1092	GLU	C	1097	SER
A	1130	ILE	B	1093	GLY	C	1098	ASN
A	1131	GLY	B	1097	SER	C	1099	GLY
A	1132	ILE	B	1098	ASN	C	1100	THR
A	1133	VAL	B	1099	GLY	C	1101	HIS
A	1134	ASN	B	1100	THR	C	1102	TRP
A	1135	ASN	B	1101	HIS	C	1103	PHE
A	1136	THR	B	1102	TRP	C	1104	VAL
A	1137	VAL	B	1103	PHE	C	1106	GLN
A	1138	TYR	B	1106	GLN	C	1107	ARG
A	1139	ASP	B	1107	ARG	C	1108	ASN
A	1140	PRO	B	1108	ASN	C	1109	PHE
A	1141	LEU	B	1109	PHE	C	1110	TYR

			B	1144	GLU			
			B	1145	LEU			
			B	1146	ASP			

A	1142	GLN	B	1110	TYR	C	1111	GLU
A	1143	PRO	B	1111	GLU	C	1112	PRO
A	1144	GLU	B	1112	PRO	C	1113	GLN
A	1145	LEU	B	1113	GLN	C	1114	ILE
A	1146	ASP	B	1114	ILE	C	1116	THR
			B	1116	THR	C	1117	THR
			B	1117	THR	C	1118	ASP
			B	1118	ASP	C	1119	ASN
			B	1119	ASN	C	1120	THR
			B	1120	THR	C	1121	PHE
			B	1121	PHE	C	1122	VAL
			B	1122	VAL	C	1123	SER
			B	1123	SER	C	1124	GLY
			B	1124	GLY	C	1125	ASN
			B	1125	ASN	C	1126	CYS
						C	1127	ASP
						C	1128	VAL
						C	1129	VAL
						C	1130	ILE
