# Supplementary material for: Single-Particle Characterization of SARS-CoV-2 Isoelectric Point and Comparison to Variants of Interest
Source: Microorganisms. 2021 Jul 28;9(8):1606. doi: 10.3390/microorganisms9081606 (PMC8401476; doi:10.3390/microorganisms9081606)
Supplement: Supplementary file 1 [file microorganisms-09-01606-s001.zip › Areo SARS COV 2 IEP Supplemental.pdf]

Single-Particle Characterization of SARS-CoV-2 Isoelectric Point and Comparison to Variants of Interest

(Supplementary Information)

Oluwatoyin Areo[1,2], Pratik U. Joshi[1,2], Mark Obrenovich[3,4,5] Moncef Tayahi[6], and Caryn L. Heldt[1,2]*

[1] Department of Chemical Engineering, Michigan Technological University, Houghton, MI USA.
[2] Health Research Institute, Michigan Technological University, Houghton, MI
[3]Louis Stokes Department of Veterans Affairs Medical Center, Department of Infectious Disease, Cleveland, OH 44106 USA.
[4]The Gilgamesh Foundation for Medical Science, Research and Education, Cleveland, OH 44116 USA
[5]Case Western Reserve University, Department of Chemistry, Cleveland, OH USA.
[6]Unversity of Cincinnati, Department of Electrical Engineering and Computer Science, Cincinnati, OH 45221
*Corresponding author: 1400 Townsend Dr., Houghton, MI 49931, USA
heldt@mtu.edu, phone 906-487-1134

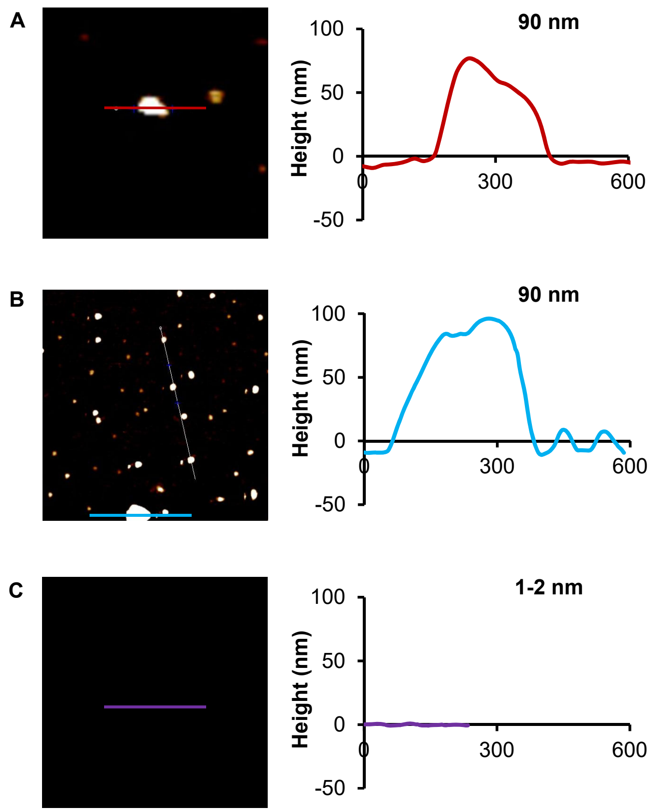

Figure S1. Topographic image and corresponding height analysis of virus and control surface. (a) SARS-CoV-2 gamma irradiated. (b) SARS-CoV-2 heat inactivated and (c) Control surface. Scan size for viruses - 3µm X 3µm, control – 500nm X 500nm

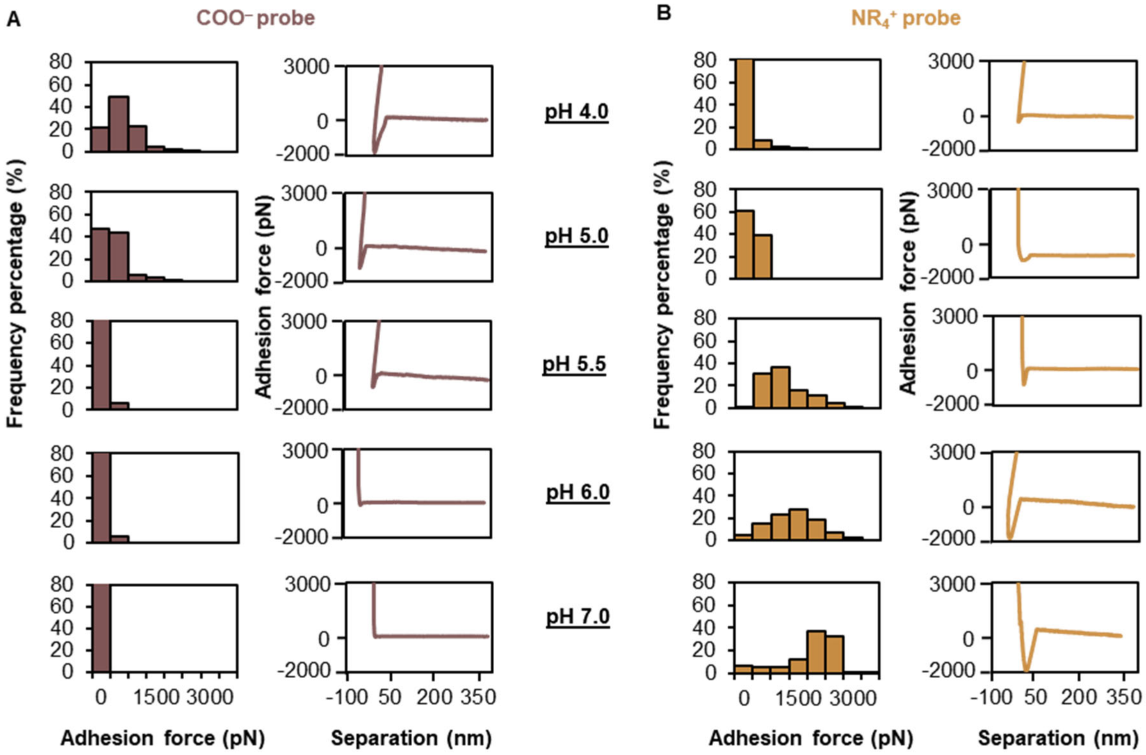

Figure S2. Adhesion and representative force–distance curves (retracted portion only) of irradiated SARS-CoV-2. (a) COO^- -terminated probe and (b) NR_4^+ -terminated probe, recorded in 20 mM citrate (pH 4-6) or phosphate buffers (pH 7). Each figure represents 450 *F–D* curves.

Table S1: Surface hydrophobicity comparison among the WT and VOI

Pango Lineage	PDB	Total Surface Residues	Hydrophobicity
WT (Natural variant)	6VSB	2454	29
B.1.1.7 (UK variant)	7LWV	2089	-146
B.1.351 (South Africa variant)	7LYQ	1992	-191

The surface residues of the WT and VOI were found by in PyMol using 'findSurfaceResidue' script. Surface hydrophobicity was calculated by summing the individual hydrophobicity values of residues assigned as per the Eisenberg hydrophobicity scale.

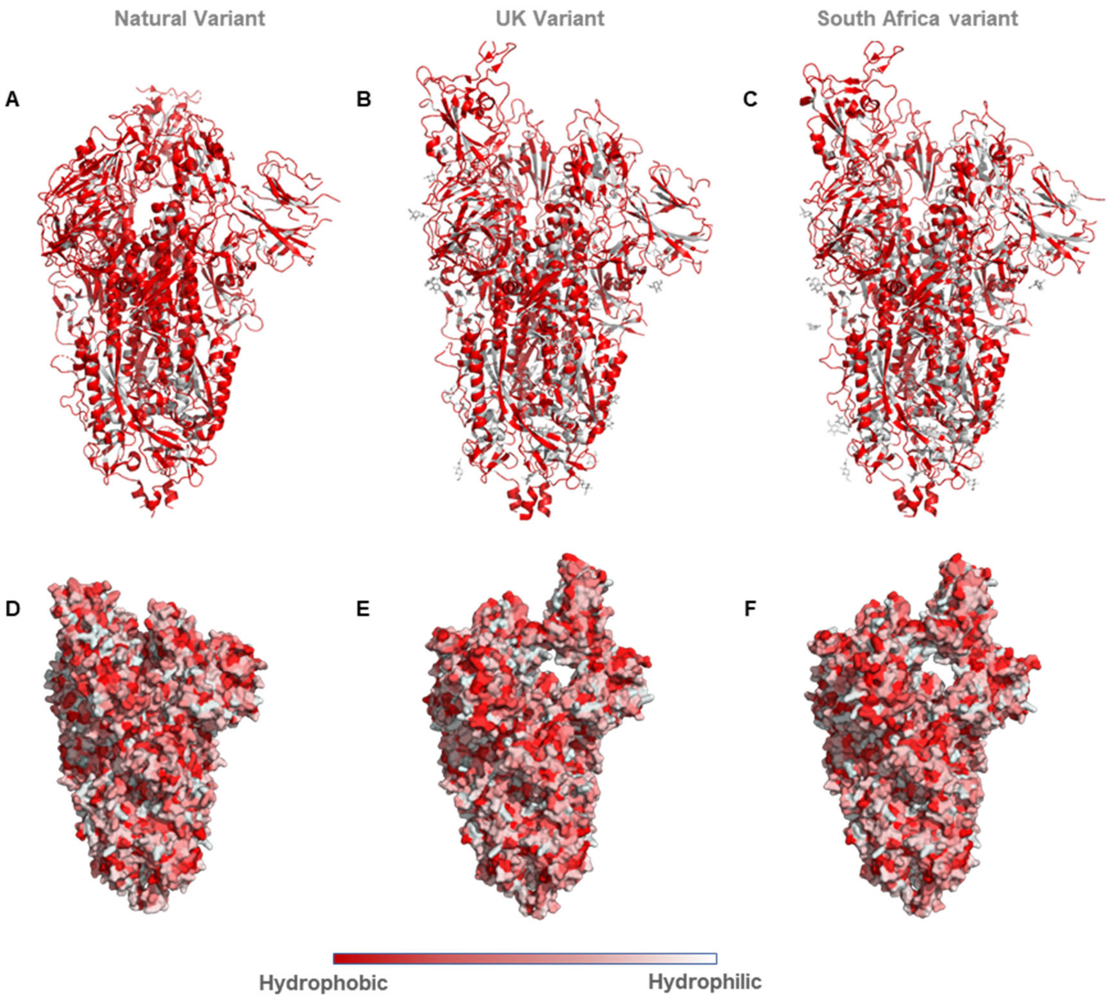

Figure S3. Surface characteristics of S-proteins of WT and VOI. (A)Surface residues (marked in red) (B) Surface hydrophobicity map of S-proteins.
